# Transcriptome Sequencing of *Listeria monocytogenes* Reveals Major Gene Expression Changes in Response to Lactic Acid Stress Exposure but a Less Pronounced Response to Oxidative Stress

**DOI:** 10.3389/fmicb.2019.03110

**Published:** 2020-01-21

**Authors:** Bienvenido W. Cortes, Annabel L. Naditz, Justin M. Anast, Stephan Schmitz-Esser

**Affiliations:** ^1^Interdepartmental Microbiology Graduate Program, Iowa State University, Ames, IA, United States; ^2^Department of Animal Science, Iowa State University, Ames, IA, United States

**Keywords:** *Listeria monocytogenes*, oxidative stress, lactic acid stress, plasmid, transcriptome, non-coding RNA, Rli47

## Abstract

*Listeria monocytogenes* is a well-characterized pathogen that represents a major threat to food safety. In this study, we examine the chromosomal and plasmid transcriptomes of two different *L. monocytogenes* strains, 6179 [belonging to sequence type (ST) 121] and R479a (ST8), in response to 30 min exposure to oxidative (0.01% hydrogen peroxide) and acid (1% lactic acid, pH 3.4) stress. The exposure to oxidative stress resulted in 102 and 9 differentially expressed (DE) genes in the chromosomal transcriptomes of 6179 and R479a, respectively. In contrast, 2280 and 2151 DE genes were observed in the respective chromosomal transcriptomes of 6179 and R479a in response to lactic acid stress. During lactic acid stress, we observed upregulation of numerous genes known to be involved in the *L. monocytogenes* stress response, including multiple members of the σ^B^ regulon, many of which have not been functionally characterized. Among these genes, homologs of *lmo2230* were highly upregulated in both strains. Most notably, the σ^B^-dependent non-coding RNA Rli47 was by far the most highly expressed gene in both 6179 and R479a, accounting for an average of 28 and 38% of all mapped reads in the respective chromosomal transcriptomes. In response to oxidative stress, one DE gene was identified in the 6179 plasmid transcriptome, and no DE genes were observed in the transcriptome of the R479a plasmid. However, lactic acid exposure resulted in upregulation of the stress response gene *clpL*, among others, on the 6179 plasmid. In R479a, a number of uncharacterized plasmid genes were upregulated, indicating a potential role in stress response. Furthermore, an average of 65% of all mapped transcriptome reads for the R479a plasmid following acid stress were mapped to an intergenic region bearing similarity to riboswitches involved in transition metal resistance. The results of this study support the conclusion that members of the σ^B^ regulon, particularly *lmo2230* and the non-coding RNA Rli47, play an integral role in the response of *L. monocytogenes* to acid stress. Furthermore, we report the first global transcriptome sequencing analysis of *L. monocytogenes* plasmid gene expression and identify a putative, plasmid-encoded riboswitch with potential involvement in response to acid exposure.

## Introduction

Food-borne pathogens present a major concern to public health, causing diseases ranging from gastroenteritis to meningitis. In some cases, these infections are severe enough to result in hospitalization or death. Therefore, preventing food-borne pathogens from contaminating foods and food production environments (FPEs) is essential. To mitigate contamination, the food production and processing industries utilize a variety of methods including strict hygiene plans, cleaning agents, disinfectants, and food additives to prevent bacterial growth. As a result, food-borne pathogens are routinely exposed to a variety of stress conditions such as high salinity and extremes in both pH and temperature. Cleaning routines and disinfecting agents can expose food-borne pathogens to heavy metals or reactive oxygen species, and the presence of preservatives such as salts and organic acids, produced by fermentation or added intentionally, present additional environmental stressors for microorganisms.

Despite these efforts, the food-borne pathogen *Listeria monocytogenes* readily persists in FPEs (Carpentier and Cerf, 2011; Ferreira et al., 2014). *L. monocytogenes* is the causative agent of listeriosis, a disease that has the potential to cause severe conditions such as meningitis, sepsis, and spontaneous abortions in susceptible individuals. Although listeriosis has a low morbidity rate, the high rates of hospitalization and mortality associated with this disease are of significant concern (Allerberger and Wagner, 2010; Scallan et al., 2011). Furthermore, the economic consequences of product recalls caused by *L. monocytogenes* contamination have been estimated to be 1.2–2.4 billion United States dollars annually (Ivanek et al., 2004), and in 2008, a single outbreak of 57 cases of listeriosis in Canada was estimated to have cost a total of 242 million Canadian dollars (Thomas et al., 2015). *L. monocytogenes* is also concerning because it is considerably more resilient to the numerous stress conditions used in FPEs to mitigate contamination than many other food-borne pathogens. As a result, *L. monocytogenes* is able to colonize niche environments in FPEs such as drainage and hard-to-clean surfaces, and therefore the complete eradication of *L. monocytogenes* from a FPE is a difficult task (Ferreira et al., 2014). In addition to persistence in FPEs themselves, the tolerance of *L. monocytogenes* to food preservatives provides the organism with a competitive advantage when growing in certain types of foods, particularly in “ready-to-eat” foods that are highly processed and have a long shelf life.

Upon exposure to environmental stress conditions found in food and FPEs, *L. monocytogenes* responds by activating a wide variety of mechanisms that confer stress tolerance (Dutta et al., 2013; Müller et al., 2013; Harter et al., 2017; Bucur et al., 2018; Hingston et al., 2019). In addition to functional characterization, multiple gene expression analyses have implicated many genes in the response of *L. monocytogenes* to temperature, oxidative, disinfectant, pH, and osmotic stresses (Oliver et al., 2009; Soni et al., 2011; Tessema et al., 2012; Casey et al., 2014; Tang et al., 2015; Hingston et al., 2017b). Until now, transcriptomic research on *L. monocytogenes* has been conducted almost exclusively on strains that lack plasmids. Previous research from our group has demonstrated that the plasmids of *L. monocytogenes* strains 6179 and R479a contribute to survival under heat, acid, salt, and oxidative stress (Naditz et al., 2019). Therefore, transcriptomic analysis may provide valuable insight into the effect of plasmids on stress tolerance and may also identify novel candidate chromosomal features permitting *L. monocytogenes* persistence in food and FPEs. Thus, this study conducted transcriptome sequencing of the plasmid-carrying strains 6179 and R479a to analyze their chromosomal and plasmid gene expression patterns after short-term exposure to oxidative and acid stress.

## Materials and Methods

### Bacterial Strains Used in This Study

*Listeria monocytogenes* 6179 is a persistent strain that belongs to ST121 and was isolated from Irish cheese in 2000 (Fox E. et al., 2011; Fox E. M. et al., 2011). It possesses a 3.01 Mbp genome and a 62.2 kbp plasmid, designated pLM6179 (Schmitz-Esser et al., 2015b). Notably, the stress survival characteristics of *L. monocytogenes* 6179 have been extensively characterized (Müller et al., 2013, 2014; Casey et al., 2014; Makariti et al., 2015; Rychli et al., 2016; Harter et al., 2017; Naditz et al., 2019). *L. monocytogenes* strain R479a is an ST8 strain isolated from smoked salmon in Denmark in 1996 where it persisted for more than 2 years in the same FPE (Fonnesbech Vogel et al., 2001; Schmitz-Esser et al., 2015a). *L. monocytogenes* R479a possesses a 2.94 Mbp genome and a 86.6 kbp plasmid, pLMR479a. Similarly to 6179, *L. monocytogenes* R479a has been subject to multiple stress survival analyses (Müller et al., 2013; Fagerlund et al., 2016; Rychli et al., 2016; Naditz et al., 2019).

### Experimental Stress Conditions

Recently, we demonstrated that plasmids pLM6179 and pLMR479a contribute to the survival of *L. monocytogenes* strains 6179 and R479a during acid (1% (vol/vol) lactic acid, pH 3.4) and oxidative stress (0.01% (vol/vol) hydrogen peroxide) (Naditz et al., 2019). To elucidate plasmid genes with potential involvement in stress response, we sequenced the transcriptomes of 6179 and R479a under the same stress conditions as applied in Naditz et al. (2019). Overnight cultures of 6179 and R479a were made by inoculating a loop of colonies into 25 mL of tryptic soy broth (TSB; Becton, Dickinson and Company) and incubating at 20°C with 200 rpm shaking for 22 h. 7.5 mL of 6179 culture and 15 mL of R479a culture were harvested and spun down at 4696 × *g* at 20°C for 10 min before pouring off the supernatants, and the 6179 and R479a pellets were then resuspended in 5 and 7.5 mL sterile 1× PBS, respectively. OD600 values were measured using a spectrophotometer (SmartSpec 3000, Bio-Rad Laboratories), and the cultures were adjusted to an OD600 of 3.5 ± 0.2. Next, 0.5 mL of adjusted overnight culture was then inoculated into 4.5 mL TSB with either a final concentration of 0.01% (vol/vol) hydrogen peroxide or 1% (vol/vol) lactic acid (pH 3.4). These tubes were then incubated at 20°C with 200 rpm shaking. To reduce the chance of significant RNA degradation, stress exposure was conducted for 30 min rather than 2 h as previously conducted (Naditz et al., 2019). Controls for both strains were created in a similar manner by inoculating 0.5 mL of adjusted overnight culture to 4.5 mL of TSB without the addition of hydrogen peroxide or lactic acid. Each condition, including the controls, was conducted in biologically independent triplicates. For the triplicates, three tubes for each condition were combined to ensure sufficiently high RNA concentrations for transcriptome sequencing.

### RNA Extraction, Library Preparation, and Sequencing

Following stress exposure for 30 min, the three tubes for each condition were combined and centrifuged at 4696 × *g* at 20°C for 3 min. Supernatants were poured off, and the pellets were each resuspended in 600 μL of Invitrogen Purelink RNA Mini Kit lysis buffer containing 1% β-mercaptoethanol. The protocol for the Invitrogen Purelink RNA Mini Kit was then followed for RNA extraction, and chemical lysis was complemented with mechanical lysis using a bead-beater (Lysing Matrix E, MP Biomedicals; Bead Mill 24 Homogenizer, Fisher Scientific). 1 μL Superase RNase inhibitor (Invitrogen) was added to each sample, and any remaining DNA was removed using the Turbo DNA-Free kit (Invitrogen) following the instructions of the manufacturer.

A PCR targeting the *prfA* gene using the primers Lip1 (5′-GAT ACA GAA ACA TCG GTT GGC-3′) and Lip2 (5′-GTG TAA TCT TGA TGC CAT CAG G-3′) (Rossmanith et al., 2006) yielded no amplified DNA, confirming the absence of *L. monocytogenes* DNA in the extracted RNA samples. PCR was conducted using the Platinum *Taq* DNA Polymerase system (Invitrogen) according to the manufacturer’s specifications. PCR cycle conditions were as follows: initial denaturation at 94°C (4 min), 35 cycles of denaturation at 94°C (30 s), annealing at 64°C (30 s), elongation at 72°C (30 s), and final elongation at 72°C (5 min). PCR products were then confirmed with agarose gel electrophoresis.

The RNA integrity of all samples was then measured using an RNA 6000 Nano chip via an Agilent 2100 Bioanalyzer (Prokaryote Total RNA Nano assay). All RNA samples had RNA integrity numbers (RIN) of at least 9.9 (Supplementary Figure S1). Samples were then sent to Microsynth (Balgach, Switzerland) for ribosomal RNA depletion using the Ribo-Zero rRNA Removal Kit (Gram-Positive Bacteria, Illumina) and stranded TruSeq library preparation. Triplicate samples were sequenced for all experimental conditions, resulting in a total of 18 samples. Single-read, 75 bp reads were generated by Microsynth using Illumina NextSeq, and read-trimming and demultiplexing were also performed by Microsynth.

### Sequence Analysis

Reads were mapped to either the *L. monocytogenes* 6179 or R479a chromosomes (HG813249.1 and HG813247.1, respectively) and plasmids (HG813250.1 and HG813248.1, respectively) using the Burrows-Wheeler aligner mem algorithm (Li and Durbin, 2010). The resulting BAM files were then imported into ReadXplorer (Hilker et al., 2014), and statistical analysis was performed using the DeSeq2 package included in ReadXplorer. DeSeq2 utilizes the Benjamini and Hochberg method for correction for multiple-testing to generate adjusted *p*-values, also called *Q*-values (Benjamini and Hochberg, 1995); *Q*-values lower than 0.05 were considered significant. Principal component analysis (PCA) was performed using R statistical software v3.5.1 (R Development Core Team, 2018) and the packages DeSeq2 v1.22.2 (Love et al., 2014) and ggplot2 v3.1.0 (Hadley, 2016). Nucleotide and amino acid alignments were done with MAFFT (Katoh et al., 2019), and shading of conserved amino acid residues was performed with Boxshade available at: https://embnet.vital-it.ch/software/BOX_form.html. RNA secondary structure predictions were generated using the RNAfold WebServer (Hofacker, 2003).

### BLAST Methods

NCBI BLAST (Wheeler et al., 2008) was used to determine homologs between the locus_tags of the 6179 and R479a chromosomes (HG813249.1 and HG813247.1, respectively) as well as homologs between the chromosomal locus_tags of the aforementioned strains and EGD-e locus_tags (NC_003210.1). Protein BLAST searches were carried out using the module NCBI-blast (2.4.0). Only those matches with 90% amino acid identity or greater were considered homologs between strains.

## Results and Discussion

### Chromosomal Gene Expression

Average chromosome coverage for each condition ranged from 165× to 232× with an average of 192× coverage for all 18 samples. On average, 97.3 and 96.4% of the total reads per sample mapped to the 6179 and R479a chromosomes, respectively (Table 1).

**TABLE 1:** Average transcriptome read statistics for all conditions^∗^.

*L. monocytogenes* strain, experimental condition	Number of reads	Chromosome coverage	Number of reads mapped to chromosome	Number of reads mapped to annotated regions chromosome	Plasmid coverage	Number of reads mapped to plasmid	Number of reads mapped to annotated regions plasmid
6179, control	8,099,939	198×	7,938,845	8,016,065	50×	41,626	34,488
6179, 0.01% hydrogen peroxide	6,636,305	162×	6,494,283	6,545,882	47×	38,829	33,355
6179, 1% lactic acid	9,442,662	223×	9,089,166	9,043,091	170×	143,073	135,899
R479a, control	8,794,964	213×	8,384,647	8,390,921	61×	71,289	64,509
R479a, 0.01% hydrogen peroxide	6,543,746	163×	6,382,773	6,495,746	53×	61,782	56,966
R479a, 1% lactic acid	7,989,763	195×	7,703,457	7,677,857	41×	48,276	45,692

*^∗^Averages are based on biological triplicates for each condition.*

**FIGURE 1:**
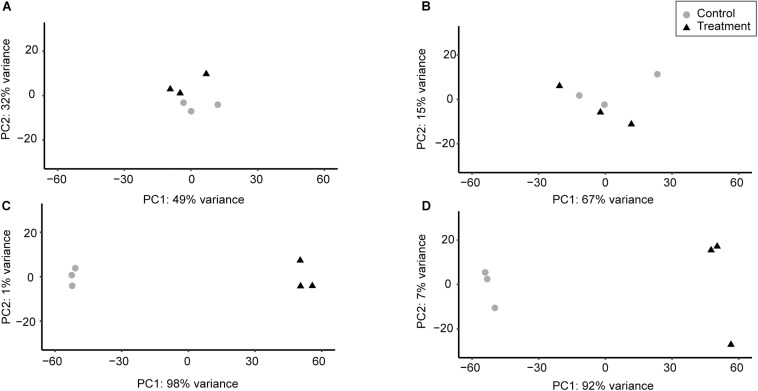
Differences between transcriptome replicates of *L. monocytogenes* strains 6179 and R479a under oxidative and acid stress conditions based on principal component analysis. For each condition, the three control replicates for each strain were compared to the three treatment replicates. **(A)** 6179 control vs. 6179 exposed to 0.01% H_2_O_2_, **(B)** R479a control vs. R479a exposed to 0.01% H_2_O_2_, **(C)** 6179 control vs. 6179 exposed to 1% lactic acid, **(D)** R479a control vs. R479a exposed to 1% lactic acid.

Principal component analysis conducted between the replicates demonstrated that for a given strain the replicates of each condition clustered closely together, indicating that there were no major disparities between replicates (Figure 1). In general, changes in the chromosomal transcription patterns of 6179 and R479a were less pronounced in response to 30 min exposure to 0.01% hydrogen peroxide than in response to 1% lactic acid. The replicates for both lactic acid exposure experiments clustered far from the control, whereas the replicates for the hydrogen peroxide experiments fell closer to the controls (Figure 1). Based on a *Q*-value of <0.05, 102 DE genes were identified in 6179 in the hydrogen peroxide treatment, and nine DE genes were identified in R479a (Table 2 and Supplementary Tables S1, S2). In contrast, lactic acid exposure resulted in differential expression of 2280 genes in 6179 and 2151 genes in R479a (Table 2 and Supplementary Tables S1, S2).

**TABLE 2:** Number of differentially expressed (DE) chromosomal and plasmid genes^∗^ in response to 1% lactic acid and 0.01% hydrogen peroxide.

*L. monocytogenes* strain, experimental condition	Number of chromosomal DE genes upregulated	Number of chromosomal DE genes downregulated	Log2fold change range (chromosome)	Number of plasmid DE genes upregulated	Number of plasmid DE genes downregulated	Log2fold change range (plasmid)
6179, 0.01% hydrogen peroxide	65	37	−1.30 to 1.14	1	0	1.11
6179, 1% lactic acid	1126	1154	−10.59 to 9.75	18	24	−4.70 to 3.15
R479a, 0.01% hydrogen peroxide	9	0	0.81 to 1.63	0	0	N/A
R479a, 1% lactic acid	1040	1111	−10.09 to 10.13	13	14	−6.14 to 3.84

*^∗^Based on a *Q*-value of <0.05.*

Further demonstrating the difference in the stress response between the two conditions, the log2fold changes of DE genes ranged from −1.30 to 1.14 in the 6179 hydrogen peroxide treatment and from 0.81 to 1.63 in the R479a hydrogen peroxide treatment. Under lactic acid stress exposure, the log2fold change ranges of 6179 and R479a were −10.59 to 9.75 and -10.09 to 10.13, respectively (Table 2 and Supplementary Tables S1, S2).

### Chromosomal Gene Expression Changes in Response to Hydrogen Peroxide Treatment

Although other studies observed pronounced transcriptional shifts (Pleitner et al., 2014) and increased expression of a functionally characterized oxidative stress survival islet (Harter et al., 2017) following 15 and 10 min exposures to oxidative stress, respectively, our study observed relatively minor transcriptomic differences even after 30 min of oxidative stress exposure. Only nine DE genes were seen in the R479a hydrogen peroxide treatment (Supplementary Table S3), although seven of these were also among the 50 DE genes of 6179 with the highest log2fold changes (Supplementary Table S4). Two of these shared DE genes are annotated as *ohrA* and *ohrR*, encoding a peroxiredoxin and its transcription factor which are involved in peroxide resistance in *Bacillus subtilis* (Fuangthong et al., 2001). In 6179, *ohrA* and *ohrR* were upregulated by log2fold changes of 1.06 and 1.04, respectively, and in R479a by respective log2fold changes of 1.02 and 0.99. In *L. monocytogenes*, transposon mutagenesis of *ohrA* resulted in decreased tolerance to hydrogen peroxide, diamide, and cumene hydroperoxide (Reniere et al., 2016). Additionally, exposure to chlorine dioxide resulted in increased levels of *ohrA* expression (Pleitner et al., 2014). Genes annotated as enzymes involved in certain steps of the citric acid cycle (*citZ* – citrate synthase, *citB* – aconitase, and *citC* – isocitrate dehydrogenase) were also upregulated in both 6179 and R479a following exposure to hydrogen peroxide. Two of these genes, *citC* and *citZ*, are part of an operon (Mittal et al., 2009) along with the upstream gene (a *lmo1568* homolog), which was upregulated as well. However, the relevance of these three citric acid cycle genes to stress response is currently unknown.

In 6179, the DE gene with the highest log2fold change (1.14) was *bilEA*, a gene involved in bile resistance (Sleator et al., 2005). However, *bilEA* was not differentially expressed in R479a. Another DE gene unique to the 6179 transcriptome in response to hydrogen peroxide exposure was *LM6179_3010*, a gene encoding a 141 amino acid protein upregulated by a log2fold change of 1.11. *LM6179_3010* shares 100% nucleotide identity with the *L. monocytogenes* EGD-e gene *lmo2230*, a known member of the σ^B^ regulon, the primary stress response regulon in *L. monocytogenes* (Raengpradub et al., 2008). LM6179_3010 and lmo2230 show 28% amino acid identity to ArsC, an arsenate reductase from *B. subtilis* (Sato and Kobayashi, 1998). However, essential cysteine amino acid residues required for function as an arsenate reductase are not conserved (Supplementary Figure S2; Bennett et al., 2001), suggesting that Lmo2230 and homologs do not function as arsenate reductases. Lmo2230 and its homologs are discussed in more detail in the section covering the lactic acid stress response below.

Also unique to the 6719 hydrogen peroxide response was the upregulation of two oxidative stress protection genes: *sodA* (*LM6179_2184*), a superoxide dismutase upregulated by a log2fold change of 0.79, and *LM6179_1300*, annotated as a glutathione peroxidase and upregulated by a log2fold change of 0.56 (Chatterjee et al., 2006; Hingston et al., 2015). The importance of the superoxide dismutase in *L. monocytogenes* stress response has been documented before (Soni et al., 2011; Bucur et al., 2018), and upregulation of the EGD-e homolog of *LM6179_1300*, *lmo0983*, was observed following exposure to chlorine dioxide (Pleitner et al., 2014). However, stress survival islet 2 (SSI-2), an insert of two genes homologous to the *Listeria innocua* genes *lin0464* and *lin0465* (*LM6179_0748 and LM6179_0749*), was not differentially expressed in 6179 despite previous implication in oxidative stress (Harter et al., 2017). The lack of significant upregulation of the SSI-2 genes under the conditions applied in our study may be explained by the differences in oxidative stress induction between the two studies. Here, 0.01% hydrogen peroxide was used to induce oxidative stress, whereas Harter et al. (2017) used 10 mM cumene hydroperoxide. The lower number of DE genes in response to hydrogen peroxide treatment in R479a compared to 6179 suggests that R479a, which belongs to a different ST than 6179, may be inherently more tolerant to hydrogen peroxide-induced oxidative stress than 6179. However, it is also possible that the 30 min oxidative stress exposure period or the concentration of hydrogen peroxide applied in this study may have been too short to induce broader changes in gene expression.

### Chromosomal Gene Expression Changes in Response to Lactic Acid Treatment

**FIGURE 2:**
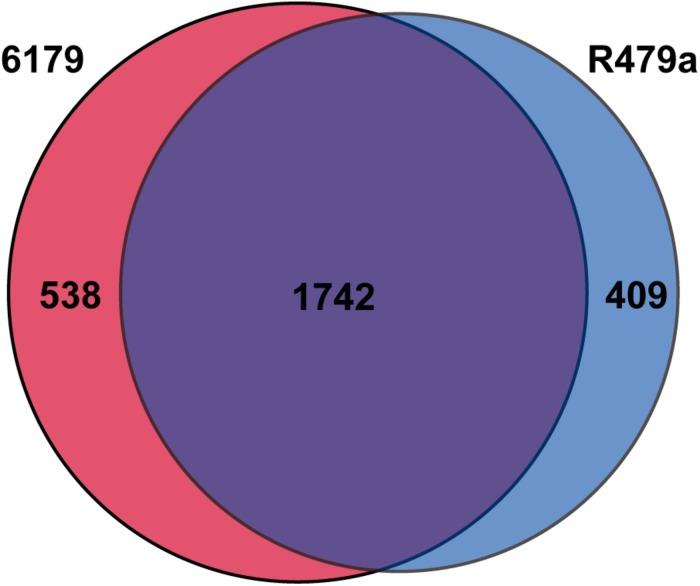
Number of unique and shared DE genes in *L. monocytogenes* strains 6179 and R479a exposed to 1% lactic acid.

As a whole, the 6179 and R479a responses to lactic acid stress were quite similar, as was expected based on their chromosomal similarity (Naditz et al., 2019). Of the genes with homologs in both strains, 1742 were differentially expressed in the transcriptomes of both 6179 and R479a (Figure 2). Only 24 of these 1742 shared DE genes had inverse expressions (upregulated in 6179 and downregulated in R479a or vice versa), thus demonstrating that the overall lactic acid stress response is highly similar between both strains. The remaining DE genes, 538 in 6179 and 409 in R479a, were unique to the response of each strain. Due to the number of DE genes, we focused further analysis on those DE genes previously implicated in acid stress response and/or with log2fold changes > 2.0.

A few notable patterns of downregulated DE genes were observed in both 6179 and R479a following lactic acid stress. Of these, the most apparent was that a large proportion of the 50 DE genes with the most negative log2fold changes encoded ribosomal proteins (23/50 in 6179 and 24/50 in R479a, Supplementary Tables S5, S6). Additional common patterns included the downregulation of genes encoding tRNAs, flagellar and motility components, chemotaxis proteins, and genes involved in replication and fatty acid biosynthesis. The downregulation of these genes suggests a decrease in general metabolic activity and motility in response to the stress conditions. In terms of general stress response, *lmo2748*, encoding the general stress protein 26 (GSP26), was upregulated by a log2fold change of 7.96 in 6179 and 7.59 in R479a. Although a role for GSP26 in osmotic stress response has been determined in *L. monocytogenes*, the same study demonstrated that a deletion of *lmo2748* does not result in deficiencies in survival under acid stress (Abram et al., 2008). Indeed, in *B. subtilis*, GSP26 is induced in response to glucose and oxygen limitation as well as to heat, osmotic, and, to a lesser degree, oxidative stress (Volker et al., 1994).

The general stress response of *L. monocytogenes* is connected to the organism’s pathogenic lifestyle by direct interactions with virulence factors. Notably, σ^B^ directly regulates the transcription of two internalins involved in initial host cell invasion, *inlA* and *inlB* (Chaturongakul et al., 2008), and a complex regulatory interaction between the σ^B^ and PrfA regulons is necessary for virulence (Chaturongakul et al., 2008; Gaballa et al., 2019). In this study, *inlA* (which possesses a premature stop codon in 6179 and is therefore annotated as two separate genes) was upregulated by log2fold changes of 7.07 and 6.95 in 6179 and 8.43 in R479a. To a lesser degree, *inlB* was also upregulated in both 6179 and R479a with log2fold changes of 4.16 and 4.18, respectively. Other notable upregulated virulence genes include *prfA* (log2fold changes of 3.70 in 6179 and 3.72 in R479a) and *hly* (log2fold changes of 1.92 in 6179 and 4.58 in R479a). These results demonstrate that even brief exposure to lactic acid at low pH is capable of inducing the expression of virulence genes in *L. monocytogenes*.

Previous studies have demonstrated that the presence of lysogenic prophages in a genome can confer beneficial effects (Edlin et al., 1977; Burns et al., 2015) including tolerance to acid stress (Wang et al., 2010). In *L. monocytogenes*, an induction of prophage gene expression has recently been reported during intracellular replication (Hain et al., 2012; Schultze et al., 2015) and for the *L. monocytogenes* 10403S A118 prophage after acid stress exposure (Ivy et al., 2012). The tRNA-Arg-TCT prophage is found in both 6179 and R479a. This prophage contained 20 DE genes in 6179 (19 upregulated with log2fold changes ranging from 0.79 to 2.98) and 11 DE genes in R479a (all upregulated ranging in log2fold change from 0.62 to 4.39). 6179 harbors two additional prophages integrated into the tRNA-Arg-CCG and tRNA-Thr-GGT loci. These had 47 DE genes (45 upregulated with log2fold changes ranging from 0.73 to 2.37) and 31 DE genes (29 upregulated with log2fold changes ranging from 1.45 to 3.29), respectively. Notably, a role in biofilm formation for the *comK* prophage found in certain *L. monocytogenes* strains including R479a has previously been supported (Verghese et al., 2011), and in this study, 29 of the 68 *comK* prophage genes in R479a were differentially expressed (28 upregulated with log2fold changes ranging from 1.10 to 4.12). However, it remains unknown whether the expression of prophage genes under stress conditions merely results in the formation of lytic phage particles or actually increases stress tolerance. Although not conclusive, the results of this study raise the possibility that the induction of these prophage genes under lactic acid stress confers beneficial effects on 6179 and R479a. However, verifying this hypothesis would require further experimentation.

Additionally, both strains analyzed in this study possess different stress survival islets (SSIs) inserted between the homologs of *lmo0443* and *lmo0449*. As mentioned above, 6179 possesses SSI-2, a two-gene insert with homology to the *L. innocua* genes *lin0464* and *lin0465*, a transcriptional regulator and protease involved in oxidative and alkaline stress (Harter et al., 2017). Corroborating the conclusion of Harter et al. (2017) that SSI-2 is not involved in acid stress, the transcriptional response of 6179 to lactic acid stress showed a significant decrease in the transcription of the *lin0464* homolog (−1.66 log2fold change) and no differential expression of the *lin0465* homolog. R479a possesses SSI-1, a five-gene insert homologous to *lmo0444*–*lmo0448*. Previous results have shown that when all five genes of SSI-1 were deleted, *L. monocytogenes* growth in pH 4.8 media was significantly decreased (Ryan et al., 2010). However, only the homologs of *lmo0444* and *lmo0445* were differentially expressed in the R479a transcriptome following lactic acid stress with log2fold changes of 0.76 and 3.76, respectively.

Two other important acid stress response systems in *L. monocytogenes* are the glutamate decarboxylase (GAD) and arginine deiminase systems (Soni et al., 2011; Bucur et al., 2018). Three genes encoding GADs (*lmo0447*, *lmo2363*, and *lmo2434*) and two genes encoding antiporters (*lmo0448* and *lmo2362*), are involved in the GAD pathway of EGD-e (Cotter et al., 2005). Notably, because 6179 lacks SSI-1, it lacks the homologs to *lmo0447* and *lmo0448* found in R479a. However, the homologs of *lmo2363*, *lmo2434*, and *lmo2362* present in both 6179 and R479a were upregulated in both strains, with *lmo2434* being upregulated by a log2fold change of 7.28 in 6179 and 8.08 in R479a. The results from this study are in line with the observation that *lmo2363* and *lmo2362* play a greater role than *lmo0447* in acid-stress survival (Cotter et al., 2001; Ryan et al., 2009). The genes encoding the arginine deiminase operon *ArcABC* (*lmo0036, lmo0037, lmo0039*, and *lmo0043*), as well as the transcriptional regulator (*lmo1367*) (Ryan et al., 2009), were all significantly upregulated in both 6179 and in R479a. Thus, as expected, two of the well-defined acid stress response mechanisms were upregulated in response to 1% lactic acid exposure.

In both 6179 and R479a, high upregulation of *mntABC* (*lmo1847, lmo1849, lmo1848*), a set of genes encoding a putative manganese transporter, was observed; the log2fold expression change ranged from 7.93 to 8.32 in 6179 and from 6.65 to 9.89 in R479a (Supplementary Tables S7, S8). Functional characterization in EGD-e has demonstrated that *mntA* is involved in host-cell penetration and macrophage survival (Reglier-Poupet et al., 2003; Bierne and Cossart, 2007). Furthermore, another gene encoding an additional putative manganese transporter, *mntH* (*lmo1424*), was highly upregulated in both strains with log2fold changes of 5.65 in 6179 and 4.32 in R479a. The high upregulation of the manganese transporters *mntABC* and *mntH* suggest that manganese transporters may also be important for *L. monocytogenes* acid stress response in addition to their described contribution to oxidative stress response and virulence.

### σ^B^ Genes

**FIGURE 3:**
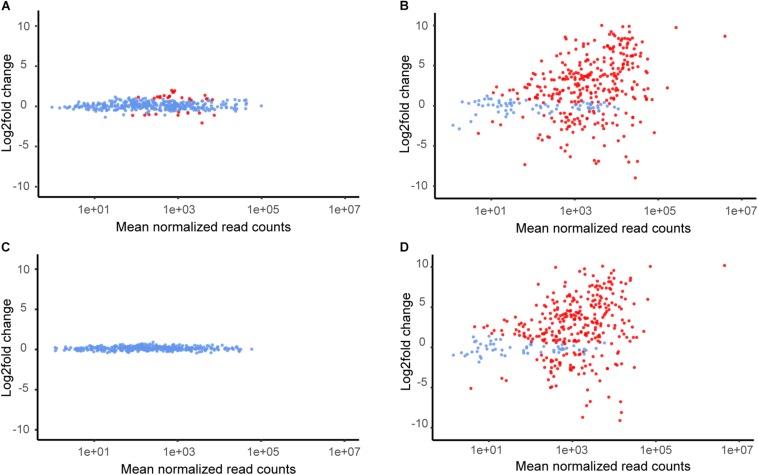
Mean of normalized read counts of all σ^B^-regulated genes plotted against log2fold expression value changes for each gene. DE genes are colored red. **(A)** *L. monocytogenes* 6179 during H_2_O_2_ stress, **(B)** *L. monocytogenes* 6179 during lactic acid stress, **(C)** *L. monocytogenes* R479a during H_2_O_2_ stress, **(D)** *L. monocytogenes* R479a during lactic acid stress.

The sigma factor σ^B^ is an important regulator of stress and virulence genes in *L. monocytogenes*. Using data from three studies (Raengpradub et al., 2008; Oliver et al., 2009; Liu et al., 2019), we created a panel of 387 genes representing the σ^B^ regulon (Supplementary Table S9); of these genes, 6179 and R479a possessed homologs to 380 and 369, respectively (Figure 3). Following the exposure to lactic acid, each strain exhibited differential expression of 311 of the σ^B^ genes found in that strain. In contrast, far fewer σ^B^ genes were differentially expressed upon exposure of either strain to hydrogen peroxide. In the case of 6179 exposed to 0.01% hydrogen peroxide, only 29 σ^B^ genes were differentially expressed, and no DE genes in the R479a oxidative stress condition were members of the σ^B^ regulon (Figure 3). It should be noted that induction of a select number of σ^B^-dependent genes was observed in *L. monocytogenes* following centrifugation before cold shock stress exposure (Chan et al., 2007). However, it is currently unknown how such an induction might affect the *L. monocytogenes* σ^B^-dependent stress response as a whole or if this induction would also occur during acid or oxidative stress. Regardless, in the case of this study, a pre-induction of σ^B^ and genes in the σ^B^ regulon during centrifugation could have potentially “primed” *L. monocytogenes* for stress exposure. While the possibility of a pre-induction of σ^B^ following the initial centrifugation step cannot be ruled out, both the control and experimental treatment samples underwent this same centrifugation step, and therefore the differences between the control and treatment transcriptomes should largely be due to stress exposure. Thus, the overall pattern of σ^B^ regulon induction suggests that the immediate response of *L. monocytogenes* strains 6179 and R479a to lactic acid exposure is at least in part mediated by σ^B^. In line with this, an upregulation of 45% of the genes from the σ^B^ regulon was recently described in a proteome study analyzing acid stress response in the *L. monocytogenes* strain Scott A (Bowman et al., 2012).

Members of the σ^B^ regulon also include a number of conserved hypothetical proteins which were differentially expressed in the experimental conditions. Additionally, many of these genes were also heavily upregulated. Following lactic acid stress, members of the σ^B^ regulon accounted for 108 and 122 DE genes encoding conserved hypothetical proteins in 6179 and R479a, respectively (the 50 most upregulated are shown in Supplementary Tables S10, S11). One of these genes, *lmo2269* (*LM6179_3049* and *LMR479a_2383* in 6179 and R479a, respectively), was the DE gene with the third highest log2fold change in 6179 (9.60) and the sixth highest log2fold change in R479a (9.89). *lmo2269* has been shown to be upregulated under heat stress in EGD-e (van der Veen et al., 2007). The numerous σ^B^ genes encoding conserved hypothetical proteins, their upregulation, and their high expression levels suggest that additional, uncharacterized σ^B^-controlled genes may be important in the *L. monocytogenes* stress response. This is in line with the observation by Oliver et al. (2010) that over 30% of the σ^B^-dependent genes shared by all strains in their study were uncharacterized, and many of them were upregulated in response to oxidative and acid stress.

Notably, the putative arsenate reductase *lmo2230* (*LM6179_3010* and *LMR479a_2344* in 6179 and R479a, respectively) was highly upregulated in both strains during lactic acid stress. This gene was the fourth highest expressed gene in 6179 and the seventh highest expressed in R479a with log2fold increases of 9.50 in 6179 and 9.85 in R479a. Multiple studies have demonstrated that *lmo2230* is a member of the σ^B^ regulon (Raengpradub et al., 2008; Oliver et al., 2009; Toledo-Arana et al., 2009; Utratna et al., 2011). These studies also showed that *lmo2230* is upregulated during early stationary phase (Raengpradub et al., 2008; Oliver et al., 2009), within the murine intestinal lumen (Toledo-Arana et al., 2009) and in response to osmotic and acid stress (Raengpradub et al., 2008; Utratna et al., 2011; Horlbog et al., 2019). Heat and cold stress have also been shown to induce *lmo2230* expression (van der Veen et al., 2007; Hingston et al., 2017a), and *lmo2230* is also upregulated after chlorine dioxide exposure (Pleitner et al., 2014).

However, in contrast to the results from the lactic acid experiments in this study, *lmo2230* expression was downregulated or only very weakly upregulated after exposure to the organic acid salts sodium diacetate and potassium lactate as well as to a combination of the two (Stasiewicz et al., 2011). Additionally, *lmo2230* was differentially expressed in response to acid stress in brain-heart infusion broth adjusted to pH 5, but the log2fold changes were much lower (1.3–2.5) than seen in this study (Tessema et al., 2012). In addition to differences in experimental conditions, the differences in upregulation of *lmo2230* could be explained by different timepoints, different *L. monocytogenes* strains, and/or the usage of a microarray compared to RNA sequencing. Although upregulation of *lmo2230* in response to stress has been observed in numerous studies (van der Veen et al., 2007; Raengpradub et al., 2008; Oliver et al., 2009; Toledo-Arana et al., 2009; Utratna et al., 2011; Pleitner et al., 2014; Hingston et al., 2017a; Horlbog et al., 2019), its function in *L. monocytogenes* remains uncharacterized. The weak amino acid identity of Lmo2230 to the arsenate reductase ArsC from *B. subtilis* might suggest a possible role in heavy metal detoxification. However, it remains true that Lmo2230 lacks conserved amino acid residues essential for function as an arsenate reductase (Supplementary Figure S2), and it is upregulated under the aforementioned stress conditions which do not involve heavy metal (arsenate or arsenic) toxicity. Taken together, these factors indicate that Lmo2230 may have a broader, yet unknown, function in *L. monocytogenes* stress response.

**FIGURE 4:**
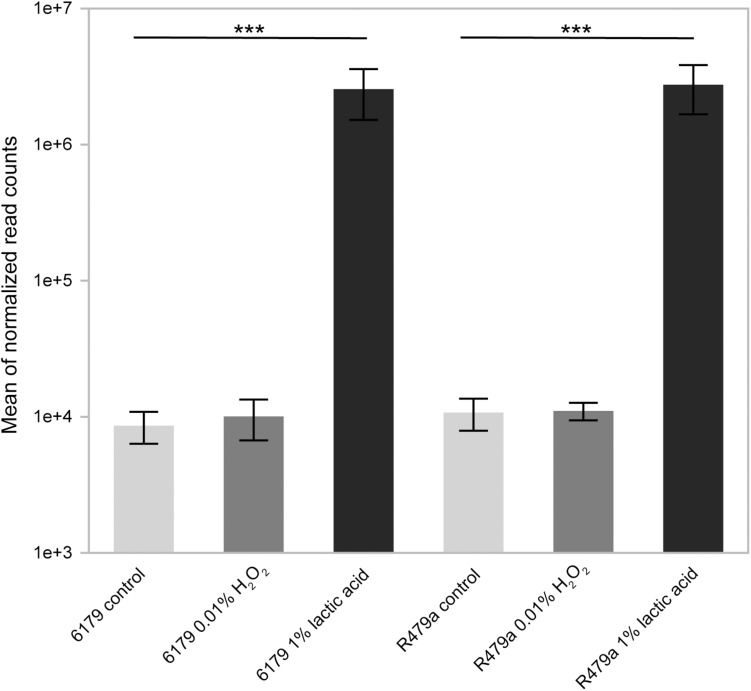
Average mean of normalized transcriptome sequencing reads mapped to the non-coding RNA Rli47 in *L. monocytogenes* strains 6179 and R479a exposed to control conditions, acid (1% lactic acid, pH 3.4), and oxidative stress (0.01% H_2_O_2_). *Q*-values (<0.001) are indicated by asterisks.

Interestingly, an extremely high percentage of all transcriptome reads from the lactic acid replicates (averaging 28 and 38% for 6179 and R479a, respectively) mapped to the non-coding RNA *rli47* (*sbrE*), a member of the σ^B^ regulon (Figure 4) (Oliver et al., 2009; Toledo-Arana et al., 2009; Mujahid et al., 2012; Liu et al., 2017). Expression of *rli47* significantly increased during exposure to lactic acid, with a log2fold change of 8.47 in 6179 and 9.97 in R479a. Recently, Marinho et al. (2019) determined that *rli47* transcripts hinder cell growth under stress conditions by suppressing translation of *ilvA*, an enzyme involved in isoleucine biosynthesis. Other studies also support the notion that *rli47* is important for response to various stressors including stationary phase (Oliver et al., 2009; Toledo-Arana et al., 2009; Mujahid et al., 2012), intracellar survival in macrophages (Mraheil et al., 2011), and oxidative stress (Mujahid et al., 2012). Taken together, our data and published gene expression and functional characterization data indicate that *rli47* likely plays a vital role in global stress response.

Here, we also observe a potential link between Rli47 and the *Listeria* adhesion protein (LAP, *lmo1634*). LAP is a bifunctional, highly conserved alcohol-acetaldehyde dehydrogenase which is essential in promoting the systemic spread of *L. monocytogenes* during infection (Pandiripally et al., 1999; Drolia et al., 2018). Previous research has demonstrated *lap* upregulation during nutrient starvation and low glucose (Jaradat and Bhunia, 2002), heme stress (Dos Santos et al., 2018), and oxygen limited environments (Burkholder et al., 2009). In our data, *lap* was significantly downregulated in both 6179 and R479a with log2fold changes of -5.10 and -2.99, respectively. Interestingly, differential expression analyses, including the one conducted in this study, consistently show a pattern of *rli47* upregulation coupled with the concurrent downregulation of *lap*. This pattern is present during stationary phase (Oliver et al., 2009; Toledo-Arana et al., 2009; Mujahid et al., 2012), the overexpression of σ^B^ (Liu et al., 2017), multiplication in the gut (Toledo-Arana et al., 2009), and in macrophages (Chatterjee et al., 2006; Mraheil et al., 2011). Furthermore, *lap* was the highest upregulated gene (29.9-fold) in an *rli47* deletion mutant (Marinho et al., 2019), suggesting that Rli47 is somehow involved in *lap* expression. The aforementioned evidence seems to implicate a relationship between LAP and Rli47 in *L. monocytogenes*, specifically that Rli47 is involved in the regulation of *lap* expression during stress conditions.

### Plasmid Gene Expression

In addition to analyzing chromosomal gene expression patterns, we sought to provide first global insights into the differential expression of *L. monocytogenes* plasmid genes under stress conditions. Although the plasmids of 6179 and R479a share 46 genes, pLM6179 and pLMR479a harbor a number of strain-specific plasmid genes, some of which are predicted to be involved in stress response (Naditz et al., 2019). Total plasmid coverage for all 18 samples ranged from 41× to 170× with an average of 71× coverage. The average of total reads per sample mapped to pLM6179 and pLMR479a were 74,509 and 60,499, respectively (Table 1).

For both strains, there were far fewer transcriptional changes following the 30 min exposure to 0.01% hydrogen peroxide than to 1% lactic acid. After hydrogen peroxide treatment, analysis identified a single DE gene in the pLM6179 plasmid transcriptome, whereas no differential expression of any pLMR479a gene was found. In contrast, the 1% lactic acid treatment resulted in 42 DE genes for pLM6179 and 27 DE genes for pLMR479a. Of these, 18 and 13 were upregulated in the 6179 and R479a plasmid transcriptomes, respectively (Table 2 and Supplementary Tables S1, S2, S12, S13). For the lactic acid treatment, log2fold changes ranged from −4.70 to 3.15 for pLM6179 and from −6.14 to 3.84 for pLMR479a (Table 2 and Supplementary Tables S1, S2, S12, S13).

### Plasmid Gene Expression Changes in Response to Hydrogen Peroxide Treatment

Interestingly, 0.01% hydrogen peroxide exposure resulted in no significant differences in plasmid gene expression between treatment and the control for R479a. In 6179, only one DE gene following 30 min exposure to 0.01% hydrogen peroxide was observed. This gene, *LM6179_RS15385*, was upregulated by a log2fold change of 1.11 and is annotated as encoding a conserved protein of unknown function. The minimal differences between the plasmid transcriptomes of the control and hydrogen peroxide treatments is in line with our chromosomal data and indicates either that 0.01% hydrogen peroxide exposure is not extremely stressful to 6179 or R479a or that the time of exposure to the stressor was insufficient to elicit major differential gene expression. It is interesting to note that our previous study showed a significant contribution of pLM6179 and pLMR479a to survival under 0.01% hydrogen peroxide, but the exposure to the stress was longer (2 h) in our previous study (Naditz et al., 2019). While not significant, the heat shock protein-encoding *clpL* gene of pLM6179 (*LM6179_RS15400*) was highly expressed in terms of read counts. An average of 47% of all reads mapped to pLM6179 mapped to *clpL* under hydrogen peroxide exposure.

### Plasmid Gene Expression Changes in Response to 1% Lactic Acid Treatment

Although pLM6179 and pLMR479a are largely distinct, they nevertheless share 46 genes, and we observed some shared expression patterns between these two plasmids in response to lactic acid exposure. First, a membrane protein of unknown function (locus_tags: LM6179_RS15475, LMR479A_RS14845) was upregulated on both plasmids. Next, two genes found on the transposon *Tn5422*, *cadA*, and *cadC*, were downregulated in 6179 and R479a. These two genes encode a cadmium-transporting protein and accessory protein, respectively (Supplementary Tables S12, S13) (Lebrun et al., 1994). The gene encoding the putative plasmid replication protein *repA* was also downregulated, indicating a decrease in plasmid replication in response to the stress conditions.

In terms of DE genes unique to each strain, in pLM6179, the most highly upregulated and expressed DE gene was *clpL*, a gene found on pLM6179 but absent from pLMR479a. *clpL* was upregulated by a log2fold change of 3.15 (Supplementary Table S12). The ClpL protein is a member of the HSP100 subgroup of heat shock proteins, and the pLM6179 ClpL protein is identical to a functionally characterized ClpL protein harbored by the *L. monocytogenes* plasmid pLM58 that functions in heat stress response (Pöntinen et al., 2017). In other studies, ClpL proteins which share more than 67% amino acid identity to the protein encoded by the pLM6179 *clpL* gene have been shown to be involved in acid stress response, tolerance to detergents and penicillin, and long-term survival in different *Streptococcus* species (Kajfasz et al., 2009; Tran et al., 2011). A ClpL homolog in *Lactobacillus reuteri* sharing 67% amino acid identity to the pLM6179 ClpL was shown to be upregulated in response to acid stress (pH 2.7) (Wall et al., 2007). This, combined with the high expression level and high upregulation of *clpL* under lactic acid stress seen in this study, further supports the hypothesis that plasmid-borne *clpL* genes in *L. monocytogenes* play a role in acid stress response. Furthermore, if *clpL* is involved in environmental stress response, then this would at least partially explain the observed trend that plasmid-cured *L. monocytogenes* 6179 is less tolerant toward lactic acid, salt, and oxidative stress (Naditz et al., 2019). Further supporting the putative role for *clpL* in *L. monocytogenes* stress response, the upregulation of plasmid-encoded *clpL* during salt and acid stress was reported recently for two *L. monocytogenes* plasmids, pLMG1-12 and pLMG1-9 (Hingston et al., 2019). Other upregulated pLM6179 DE genes included mainly uncharacterized genes, a pair of putative toxin-antitoxin genes (*LM6179_RS15455* and *LM6179_RS15450*), and a gene (*LM6179_RS15380*) found on many *L. monocytogenes* plasmids that is putatively involved in UV protection or DNA repair (Kuenne et al., 2010).

In response to 1% lactic acid treatment, the upregulated pLMR479a DE genes primarily consisted of genes encoding uncharacterized proteins for which no potential function can be inferred based on sequence similarity (Supplementary Table S13). However, a putative multicopper oxidase gene (*LMR479a_RS15145*) was also upregulated. The protein encoded by *LMR479a_RS15145* shows more than 95% amino acid identity to a homolog in *Staphylococcus aureus* ATCC12600 which has been demonstrated to be involved in copper resistance and oxidative stress response (Sitthisak et al., 2005). However, the putative multicopper oxidases on pLMR479a and on other *Listeria* plasmids encode 386 amino acid proteins, which are shorter than the *S. aureus* homologs (462 amino acids). Recently, an identical homolog of the putative multicopper oxidase was also found to be upregulated under mild acid stress (pH 5) in the *L. monocytogenes* plasmid pLMG1-12 (Hingston et al., 2019).

Of significant interest was that the majority (65% on average) of the pLMR479a reads during lactic acid stress were mapped to an intergenic region (position 81657 to 81765) between locus_tags *LMR479a_RS15240* and *LMR479a_RS15245* (Supplementary Figure S3). Additionally, expression of this intergenic region was significantly upregulated by a log2fold change of 3.27. This region shows 56% nucleotide identity to the RFAM family RF02683, a recently described family of nickel–cobalt (NiCo) riboswitches known to be involved in transition metal tolerance (Furukawa et al., 2015). In *Firmicutes*, such as *Clostridiales* and *Erysipelotrichaceae*, these riboswitches bind to ions such as zinc, copper, nickel, and cobalt, and this binding activity is utilized in the detection of and response to toxic concentrations of transition metals (Furukawa et al., 2015). The putative riboswitch identified on pLMR479a shares 70% nucleotide identity and 90% percent coverage with both the functionally characterized *Erysipelotrichaceae* bacterium 3_1_53 NiCo riboswitch and the *Clostridium scindens* ATCC 35704 NiCo riboswitch (Furukawa et al., 2015; Supplementary Figures S4, S5). RNA secondary structural predictions revealed that the putative riboswitch from pLMR479a possessed a similar secondary structure to those of the functionally characterized NiCo riboswitches showing the four base-paired regions described by Furukawa et al. (2015) (Supplementary Figure S6).

Furukawa et al. (2015) also identified homologs of these NiCo riboswitches in *Listeria*. Interestingly, all homologs in *Listeria* identified by Furukawa et al. (2015) were found on plasmids, such as the homolog harbored on *L. monocytogenes* strain 08-5578 plasmid pLM5578. In contrast, the riboswitch homologs in *Erysipelotrichaceae* bacterium 3_1_53 and *C. scindens* are located on the chromosome. Notably, while the putative pLMR479a riboswitch shows high similarity to the characterized riboswitch homologs in *Erysipelotrichaceae* bacterium 3_1_53 and *C. scindens*, the transporter genes located upstream of these riboswitches show no similarity. All of the characterized NiCo riboswitches identified by Furukawa et al. (2015), including the putative *Listeria* riboswitches, are located upstream of putative heavy or transition metal transporters that correspond with the transition metal ions bound by the associated riboswitch. Similarly, the characterized riboswitches in *Erysipelotrichaceae* bacterium 3_1_53 and *C. scindens* are associated with a NiCo transporter. In pLMR479a, the putative riboswitch is located upstream of a putative zinc-transporter (*LMR479a_RS15240*), showing 41% amino acid identity to ZosA from *B. subtilis*, a zinc transporter important in oxidative stress response (Gaballa and Helmann, 2002). However, it should be noted that the *zosA*-like gene was not a DE gene under lactic acid stress applied here. The pLMR479a ZosA-like transporter is a 627 amino acid protein and belongs to the P-type ATPase superfamily 3.A.3 of the transporter classification database (Saier et al., 2016), whereas the transporters downstream of *Erysipelotrichaceae* bacterium 3_1_53 and *C. scindens* riboswitches are 308 amino acid residues in length and belong to the cation diffusion facilitator (CDF) family 2.A.4. This suggests that although transition metal-binding riboswitches may be similar, their associated transition metal transporters can be highly variable. BlastN searches against NCBI Genbank using the putative pLMR479a riboswitch as query revealed 44 identical homologs in different *L. monocytogenes* plasmids (Supplementary Table S14), suggesting that homologs of these putative riboswitches are found on a number of *L. monocytogenes* plasmids. Here, we provide the first gene expression data for this putative, plasmid-borne riboswitch in *L. monocytogenes.* This data shows that transcription of this riboswitch accounts for up to 73% of the transcripts of pLMR479a during 1% lactic acid stress. In the future, the potential function of this putative riboswitch in acid stress response and its regulation of the upstream ZosA-like transporter will need to be verified.

## Conclusion

Here, we provide detailed insights into the short-term stress response of two *L. monocytogenes* strains, 6179 and R479a, to oxidative and acid stress. In particular, the lactic acid exposure resulted in striking changes in chromosomal gene expression. Our results provide more evidence for the importance of σ^B^-regulated genes in stress response, including *lmo2230* and its homologs, and show massive transcription of the non-coding RNA *rli47*. Additionally, our results demonstrate that the overall chromosomal responses to the aforementioned stress conditions are highly similar between 6179 and R479a. This study also provides the first in-depth analyses of plasmid gene expression in *L. monocytogenes*. Our results from two *L. monocytogenes* strains carrying distinct plasmids show that known and putative plasmid-encoded stress response genes such as *clpL* and the multi-copper oxidase are highly expressed and upregulated in response to stress conditions. We also reveal evidence in support of an important role of a putative, uncharacterized riboswitch in lactic acid stress response in pLMR479a based on its high expression level and significant upregulation during acid stress.

## Data Availability Statement

The raw sequencing data were deposited at the NCBI Sequence Read Archive under Bioproject No. PRJNA563702.

## Author Contributions

SS-E, BC, and AN were involved in study design and conceived the experiments. BC and AN performed the experiments. BC, AN, JA, and SS-E analyzed the data, wrote the manuscript, and approved the final manuscript.

## Conflict of Interest

The authors declare that the research was conducted in the absence of any commercial or financial relationships that could be construed as a potential conflict of interest.
