# Supplementary material for: Transcriptome Sequencing of Listeria monocytogenes Reveals Major Gene Expression Changes in Response to Lactic Acid Stress Exposure but a Less Pronounced Response to Oxidative Stress
Source: Front Microbiol. 2020 Jan 21;10:3110. doi: 10.3389/fmicb.2019.03110 (PMC6985202; doi:10.3389/fmicb.2019.03110)
Supplement: Supplementary file 1 [file Data_Sheet_1.PDF]

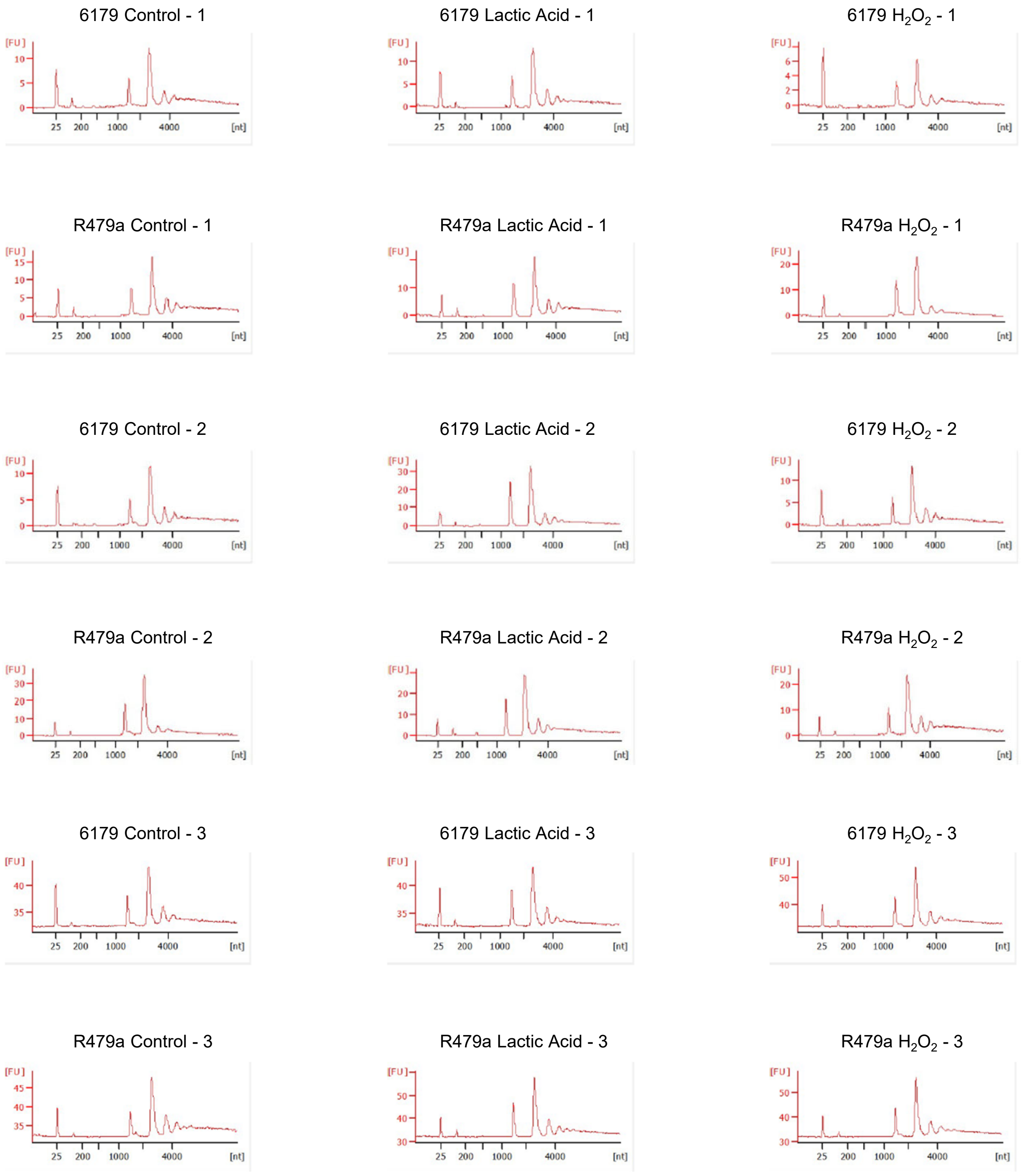

Supplementary Figure S1. Electropherogram results of extracted RNA samples. The RNA integrity of the samples was measured using an RNA 6000 Nano chip via an Agilent 2100 Bioanalyzer (Prokaryote Total RNA Nano assay).

```
lmo2230_EGDe        1 MTQKLIYFL-SQTHIRSAIAEAWAKRLSLSNVKFISGSWHKSKSTPFIAEALNEFAIEPP
LM6179_3010_6179    1 MTQKLIYFL-SQTHIRSAIAEAWAKRLSLSNVKFISGSWHKSKSTPFIAEALNEFAIEPP
LMR479A_2344_R479   1 MTQKLIYFL-SQTHIRSAIAEAWAKRLSLSNVKFISGSWHKSKSTPFIAEALNEFAIEPP
P45947_ARSC         1 MENKIIYFLCTGNSCRSQMAEGWAKQYLGDEWKVYSAGIEAHGLNPNAVKAMKEVGIDIS
consensus           1 *..*.**** .....**..**.***.......*..*.........*....*..*..*...

lmo2230_ EGDe      60 ESLSYSPSSELLADADLIVTIYDSAHETAPKFPANIQEKIIYWDIDDPEQEIAL-PQKWA
LM6179_3010_6179   60 ESLSYSPSSELLADADLIVTIYDSAHETAPKFPANIQEKIIYWDIDDPEQEIAL-PQKWA
LMR479A_2344_R479  60 ESLSYSPSSELLADADLIVTIYDSAHETAPKFPANIQEKIIYWDIDDPEQEIAL-PQKWA
P45947_ARSC        61 NQTSDIIDSDILNNADLVVTLCGDAADKCPMTPPHVKRE--HWGFDDPARAQGTEEEKWA
consensus          61 ...*....*..*..***.**....*....*..*.........*..***...... ..***

lmo2230_ EGDe      119 SYQEVCDNIALSVKNLEHVLIEA
LM6179_3010_6179   119 SYQEVCDNIALSVKNLEHVLIEA
LMR479A_2344_R479 119 SYQEVCDNIALSVKNLEHVLIEA
P45947_ARSC       119 FFQRVRDEIGNRLKEFAET--GK
consensus         121 ..*.*.*.*....*.........
```

Supplementary Figure S2. Amino acid alignment of putative arsenate reductase from *L. monocytogenes* with the arsenate reductase ArsC from *Bacillus subtilis*. The alignment was done with MAFFT available at: https://mafft.cbrc.jp/alignment/server/, shading of conserved amino acid residues was performed with Boxshade available at: https://embnet.vital-it.ch/software/BOX_form.html. Asterisks indicate identical positions, dots indicate similar positions. Amino acid residues essential for function od ArsC from *Bacillus subtilis* based on (Bennett et al., Proc Natl Acad Sci U S A. 2001;98(24):13577-82) are highlighted in red. Abbreviations : lmo2230_ EGDe: *L. monocytogenes* EGDe, LM6179_3010_6179: *L. monocytogenes* 6179, LMR479A_2344_R479: *L. monocytogenes* R479a, and P45947_ARSC: *Bacillus subtilis* ArsC.

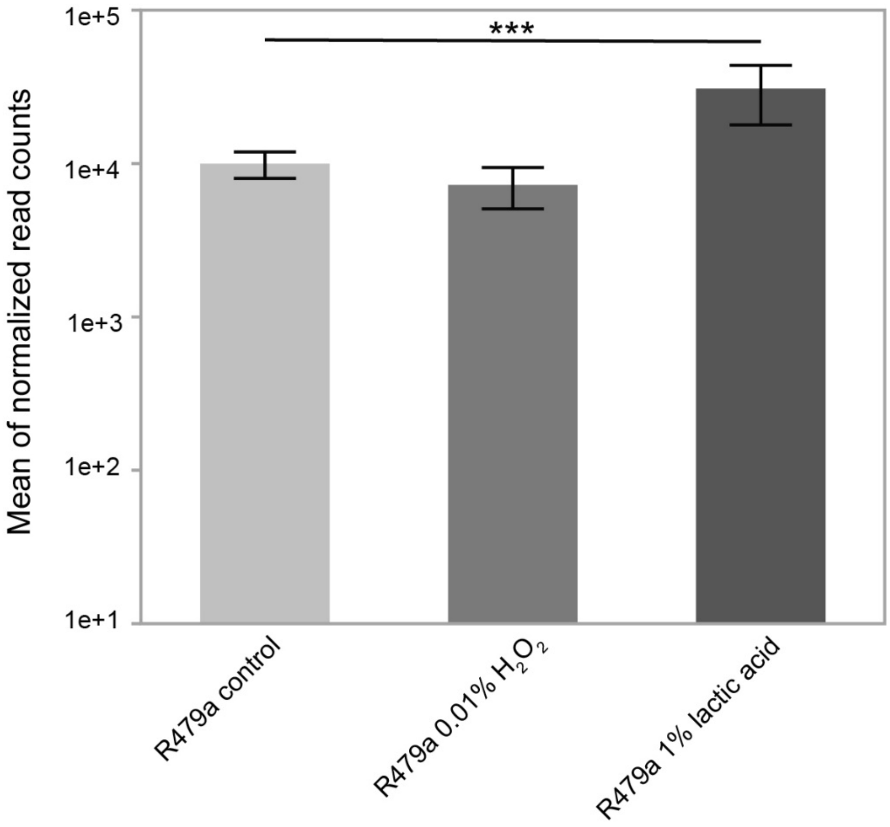

Supplementary Figure S3. Average mean of normalized transcriptome sequencing reads mapped to the noncoding RNA in *L. monocytogenes* strain R479a plasmid exposed to control conditions, oxidative stress (0.01% H_2O_2), and acidic stress (1% lactic acid). Q-values (<0.001) are indicated by asterisks.

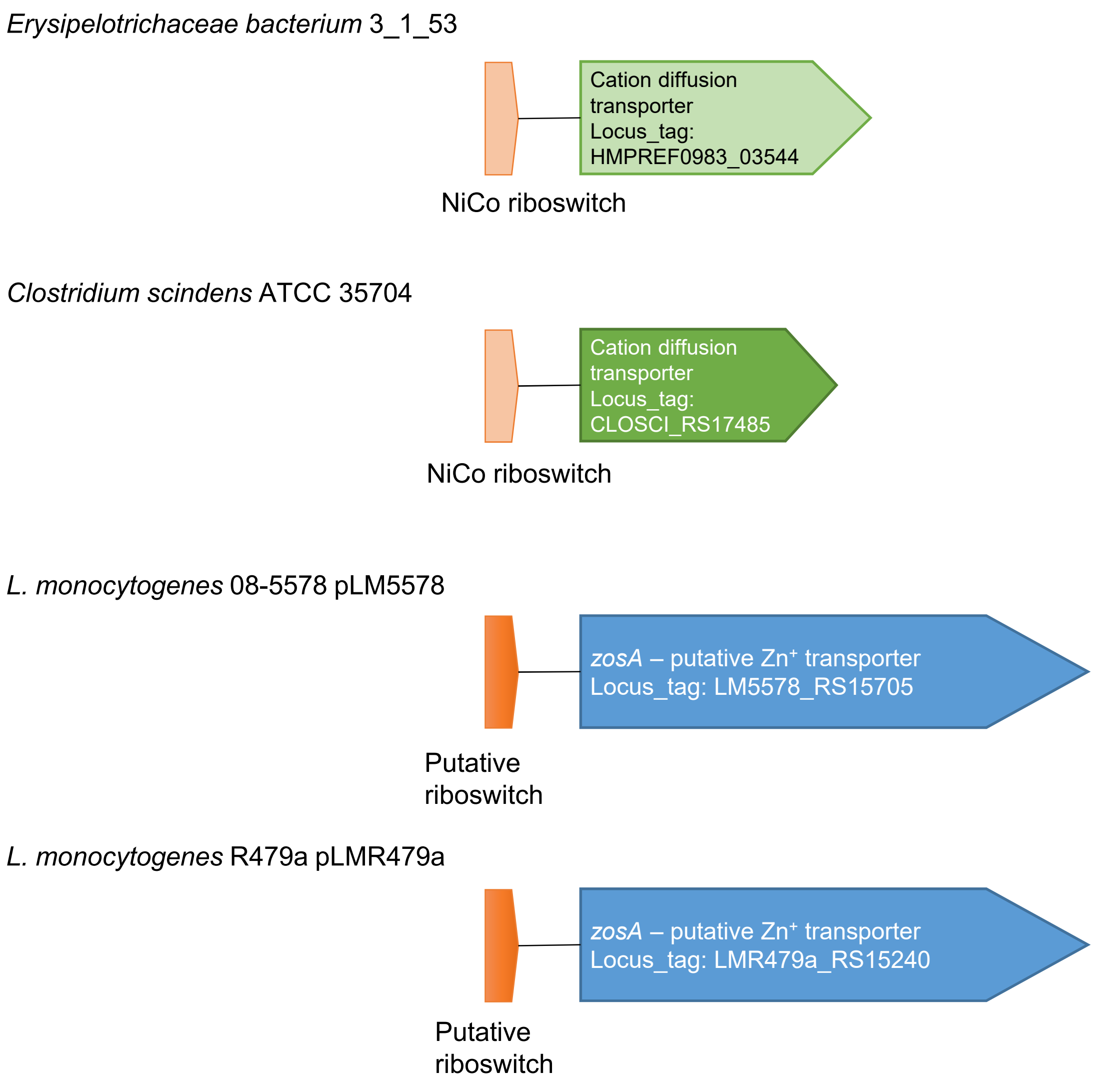

Supplementary Figure S4. Visual representation of the genomic organization of functionally characterized NiCo riboswitches from *Erysipelotrichaceae bacterium* 3_1_53 and *Clostridium scindens* ATCC 35704 and putative riboswitches found on *Listeria* plasmids.

```
C_scindens     1 ACAGUACAAACUGAGCAGGCGAUGAACCUUUA-----GGUCAUGGGGCCGGGCCGCUU--
Eba            1 ACAGUACAAACUGAGCAGGCAAUGACCAGAGC-----GGUCAUGCAGCCGGGCUGCGA--
pLM5578        1 UCAUUGUGAUCUGAACAGGCGGUGAACGUAACACGAGGUUCAUGCAGCUGGGCUGCAAUU
pLMR479a       1 UCAUUGUGAUCUGAACAGGCGGUGAACGUAACACGAGGUUCAUGCAGCUGGGCUGCAAUU
consensus      1  ** *. .* ****.*****..***.*..........* *****..** ****.**....

C_scindens    54 --UUGUGGCAGCAGAUUGCAAUUUC----AGCACAUCUGUGGGACAGUU
Eba           54 --AAGCGGCAACAGAUGAUUACAC------GCACAUCUGUGGGACAGUU
pLM5578       61 AUUUGCGGCAGCAGACUAUGUAUUCUAAGGGCAUAUCUGUGGGACAGUU
pLMR479a      61 AUUUGCGGCAGCAGACUAUGUAUUCUAAGGGCAUAUCUGUGGGACAGUU
consensus     61 ....*.****.**** .... .........*** ***************
```

Supplementary Figure S5. Nucleotide alignment of functionally characterized NiCo riboswitches and putative riboswitch from *Listeria monocytogenes* plasmid pLMR479a. The alignment was done with MAFFT available at: https://mafft.cbrc.jp/alignment/server/, shading of conserved nucleotide residues was performed with Boxshade available at: https://embnet.vital-it.ch/software/BOX_form.html. Asterisks indicate identical positions, dots indicate similar positions. Nucleotide positions conserved in more than 97% of the sequences are highlighted in blue, positions conserved in more than 90% of the sequences are highlighted in teal. Conservation of nucleotide positions is based on (Furukawa et al., Mol Cell 2015; 57:1088-1098). Abbreviations : C_scindens: *C. scindens* (Accession number: NZ_ACTJ00000000.1), Eba: *Erysipelotrichaceae* bacterium 3_1_53 (Accession number: NZ_ABFY00000000.2), pLM5578: L. monocytogenes 08-5578 (Accession number: GCA_ 000093125.2), and pLMR479a: *L. monocytogenes* R479a.

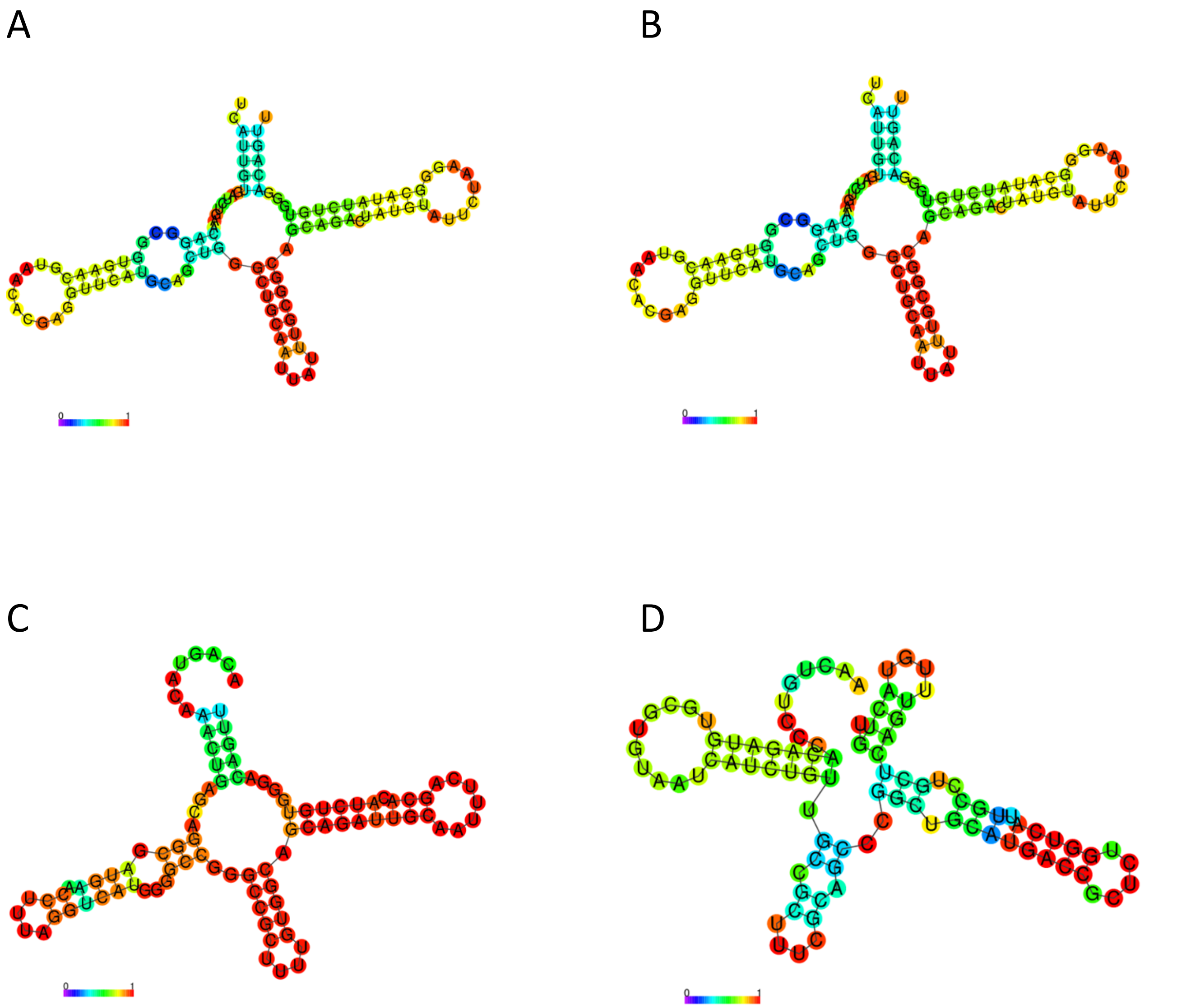

Supplementary Figure S6. Predicted secondary structure for functionally characterized NiCo riboswitches and putative riboswitches from pLMR479a and pLM5578.

Predicted secondary structures of the putative riboswitches from A) *L. monocytogenes* pLMR479a, B) *L. monocytogenes* pLM5578, and the functionally characterized NiCo riboswitches from C) *C. scindens* ATCC35704, and D) *Erysipelotrichaceae* bacterium 3_1_53. Secondary structure predictions were generated using the RNAfold WebServer (http://rna.tbi.univie.ac.at//cgi-bin/RNAWebSuite/RNAfold.cgi) (Hofacker, Nucleic Acids Res 2003; 31(13): 3429-3431).
