# Supplementary material for: Transcriptome Sequencing of Listeria monocytogenes Reveals Major Gene Expression Changes in Response to Lactic Acid Stress Exposure but a Less Pronounced Response to Oxidative Stress
Source: Front Microbiol. 2020 Jan 21;10:3110. doi: 10.3389/fmicb.2019.03110 (PMC6985202; doi:10.3389/fmicb.2019.03110)
Supplement: Supplementary file 2 [file Data_Sheet_2.PDF]

Supplementary Table S3: Chromosomal DE genes in the *L. monocytogenes* R479a transcriptome after 30 min exposure to 0.01% hydrogen peroxide. DE genes which are also upregulated DE genes in 6179 under hydrogen peroxide treatment are highlighted in bold.

L. monocytogenes **R479a locus_tag**	**Gene**	**Product**	***L. monocytogenes*** **EGDe locus_tag**	***L. monocytogenes*** **6179 locus_tag**	**log2foldchange**	**Q value**
**LMR479A_1738**	***citB***	**aconitate hydratase (aconitase)**	**lmo1641**	**LM6179_2393**	**1.63**	**2.38E-09**
**LMR479A_1661**	***citZ***	**citrate synthase II**	**lmo1567**	**LM6179_2318**	**1.21**	**1.69E-07**
**LMR479A_1662**	***ytwI***	**putative integral membrane protein**	**lmo1568**	**LM6179_2319**	**1.30**	**2.65E-07**
**LMR479A_1660**	***citC***	**isocitrate dehydrogenase**	**lmo1566**	**LM6179_2317**	**1.33**	**4.94E-06**
LMR479A_1994	*xpt*	xanthine phosphoribosyltransferase	lmo1885	LM6179_2654	1.07	7.17E-05
**LMR479A_0866**	***artR***	**High affinity arginine ABC transporter (ATP-binding protein)**	**lmo0848**	**LM6179_1160**	**0.81**	**4.55E-03**
LMR479A_1993	*pbuX*	xanthine permease	lmo1884	LM6179_2653	0.88	8.25E-03
**LMR479A_2312**	***ohrA***	**peroxiredoxin**	**lmo2199**	**LM6179_2978**	**1.02**	**1.38E-02**
**LMR479A_2313**	***ohrR***	**transcriptional regulator sensing organic peroxides**	**lmo2200**	**LM6179_2979**	**0.99**	**1.55E-02**

Supplementary Table S4: The 50 most upregulated chromosomal DE genes in the *L. monocytogenes* 6179 transcriptome after 30 min exposure to 0.01% hydrogen peroxide. Genes that are part of the Sigma B regulon are highlighted in blue. DE genes which are also upregulated DE genes in R479a under hydrogen peroxide treatment are highlighted in bold.

L. monocytogenes **6179 locus_tag**	**Gene**	**Product**	***L. monocytogenes*** **EGDe locus_tag**	***L. monocytogenes*** **R479a locus_tag**	**log2foldchange**	**Q value**
LM6179_2165	*bilEA*	BilEA	lmo1421	LMR479a_1510	1.143	1.90E-06
LM6179_3010		putative Arsenate reductase	lmo2230	LMR479a_2344	1.113	5.71E-05
**LM6179_2317**	***citC***	**isocitrate dehydrogenase**	**lmo1566**	**LMR479a_1660**	**1.074**	**1.32E-05**
**LM6179_2978**	***ohrA***	**peroxiredoxin**	**lmo2199**	**LMR479a_2312**	**1.056**	**1.82E-04**
**LM6179_2979**	***ohrR***	**transcriptional regulator sensing organic peroxides**	**lmo2200**	**LMR479a_2313**	**1.041**	**5.31E-05**
LM6179_2390	*yocD*	putative carboxypeptidase	lmo1638	LMR479a_1735	1.018	9.08E-06
LM6179_2166		conserved membrane protein of unknown function	lmo1422	LMR479a_1511	1.014	3.99E-05
LM6179_1818		conserved protein of unknown function	lmo2454	LMR479a_2579	1.012	1.45E-07
**LM6179_2319**	***ytwI***	**putative integral membrane protein**	**lmo1568**	**LMR479a_1662**	**1.005**	**4.57E-08**
**LM6179_2318**	***citZ***	**citrate synthase II**	**lmo1567**	**LMR479a_1661**	**0.980**	**7.80E-05**
**LM6179_2393**	***citB***	**aconitate hydratase (aconitase)**	**lmo1641**	**LMR479a_1738**	**0.968**	**1.63E-03**
LM6179_2392		conserved membrane protein of unknown function	lmo1640	LMR479a_1737	0.914	2.43E-03
LM6179_0248		conserved protein of unknown function	lmo2828	LMR479a_2966	0.903	1.32E-03
LM6179_3001		conserved protein of unknown function	lmo2221	LMR479a_2335	0.861	2.81E-06
LM6179_2391		DNA-3-methyladenine glycosylase I	lmo1639	LMR479a_1736	0.849	8.53E-03
LM6179_misc_RNA_43		LhrC			0.833	3.29E-03
LM6179_2637	*ppdK*	Pyruvate, phosphate dikinase	lmo1867	LMR479a_1977	0.830	7.16E-04
LM6179_2745	*dinB*	DNA polymerase IV	lmo1975	LMR479a_2085	0.829	9.17E-04
LM6179_1716	*uvrA*	excinuclease ABC (subunit A)	lmo2488	LMR479a_2613	0.824	1.66E-05
LM6179_2184	*sodA*	superoxide dismutase	lmo1439	LMR479a_1528	0.793	8.02E-03
LM6179_2635	*ccpN*	negative regulator of gluconeogenesis	lmo1865	LMR479a_1975	0.783	2.29E-04
LM6179_3002		conserved protein of unknown function	lmo2222	LMR479a_2336	0.781	8.99E-04
LM6179_0112	*dhaM*	PTS-dependent dihydroxyacetone kinase, phosphotransferase subunit dhaM	lmo2697	LMR479a_2835	0.771	3.61E-03
LM6179_1715	*uvrB*	excinuclease ABC (subunit B)	lmo2489	LMR479a_2614	0.771	3.88E-04
LM6179_2044	*yneA*	Cell division suppressor protein YneA	lmo1303	LMR479a_1387	0.757	4.01E-02

LM6179_0111	*dhaL*	PTS-dependent dihydroxyacetone kinase, ADP-binding subunit dhaL	lmo2696	LMR479a_2834	0.733	1.55E-02
LM6179_1447		conserved protein of unknown function	lmo1140	LMR479a_1161	0.710	6.45E-04
LM6179_0110	*dhaK*	dihydroxyacetone kinase, N-terminal domain	lmo2695	LMR479a_2833	0.709	1.59E-02
LM6179_0907		conserved protein of unknown function	lmo0602	LMR479a_0614	0.684	1.19E-02
LM6179_2042	*lexA*	transcriptional repressor of the SOS regulon	lmo1302	LMR479a_1386	0.678	3.21E-03
LM6179_2142	*recA*	multifunctional SOS repair factor	lmo1398	LMR479a_1486	0.677	1.27E-02
LM6179_1912		conserved protein of unknown function			0.664	3.30E-02
LM6179_1628		conserved protein of unknown function	lmo2571	LMR479a_2699	0.661	3.82E-02
LM6179_2934	*BDS*	Alkyl/aryl-sulfatase BDS1	lmo2157	LMR479a_2269	0.639	4.56E-02
LM6179_2990		conserved protein of unknown function	lmo2210	LMR479a_2324	0.624	1.19E-02
LM6179_2636	*yqfL*	positive regulator of gluconeogenesis	lmo1866	LMR479a_1976	0.622	3.53E-02
LM6179_2871		Transcriptional regulator	lmo2100	LMR479a_2211	0.621	9.81E-03
LM6179_2821		conserved protein of unknown function	lmo2050	LMR479a_2161	0.612	3.46E-02
LM6179_2550	*exoA*	apurinic/apyrimidinic endonuclease	lmo1782	LMR479a_1890	0.601	2.89E-02
LM6179_0156		conserved protein of unknown function	lmo2742	LMR479a_2879	0.600	3.85E-02
LM6179_1107	*ywnB*	putative oxidoreductase	lmo0794	LMR479a_0813	0.585	1.48E-02
LM6179_3046		Fumarylacetoacetate hydrolase family protein	lmo2266	LMR479a_2380	0.577	1.53E-02
LM6179_0514	*cysK*	cysteine synthase	lmo0223	LMR479a_0232	0.576	1.48E-02
LM6179_0867	*hisI*	fragment of phosphoribosyl-AMP cyclohydrolase; phosphoribosyl-ATP pyrophosphohydrolase (part 1)	lmo0562	LMR479a_0574	0.573	3.13E-02
LM6179_0962		Acetyltransferase	lmo0652	LMR479a_0669	0.572	1.47E-04
LM6179_0447		conserved protein of unknown function	lmo0157	LMR479a_0166	0.567	6.45E-04
LM6179_1300	*gpo*	Glutathione peroxidase	lmo0983	LMR479a_1009	0.562	4.31E-02
LM6179_2264		conserved protein of unknown function	lmo1518	LMR479a_1611	0.558	6.95E-04
LM6179_1625		conserved protein of unknown function	lmo2574	LMR479a_2702	0.545	2.43E-03
LM6179_2122	*lisK*	LisK	lmo1378	LMR479a_1466	0.545	1.24E-02

Supplementary Table S5: The 50 most downregulated chromosomal DE genes in the *L. monocytogenes* 6179 transcriptome after 30 min exposure to 1% lactic acid. Genes that are part of the Sigma B regulon are highlighted in blue. DE genes which are also downregulated DE genes in R479a under lactic acid treatment are highlighted in bold.

L. monocytogenes **6179 locus_tag**	**Gene**	**Product**	***L. monocytogenes*** **EGDe locus_tag**	***L. monocytogenes*** **R479a locus_tag**	**log2foldchange**	**Q value**
**LM6179_1852**		**Cation efflux family protein**	**lmo2423**	**LMR479a_2546**	**-10.59**	**1.54E-70**
**LM6179_1681**	***yocH***	**Cell wall-binding protein yocH**	**lmo2522**	**LMR479a_2648**	**-10.12**	**2.35E-154**
**LM6179_0443**	***znuA***	**Zn(II)-binding lipoprotein**	**lmo0153**	**LMR479a_0162**	**-9.47**	**4.57E-09**
**LM6179_0986**		**conserved protein of unknown function**	**lmo0675**	**LMR479a_0693**	**-8.57**	**4.91E-21**
**LM6179_0476**		**conserved protein of unknown function**	**lmo0186**	**LMR479a_0195**	**-8.33**	**1.09E-240**
**LM6179_1697**	***ftsE***	**cell-division ABC transporter (ATP-binding protein)**	**lmo2507**	**LMR479a_2632**	**-8.17**	**7.94E-234**
**LM6179_1698**	***ftsX***	**cell-division ABC transporter**	**lmo2506**	**LMR479a_2631**	**-8.15**	**2.28E-270**
**LM6179_2555**		**conserved protein of unknown function**		**LMR479a_1895**	**-7.87**	**6.44E-06**
**LM6179_1700**		**Peptidase**	**lmo2504**	**LMR479a_2629**	**-7.83**	**1.33E-190**
**LM6179_1699**	***spl***	**P45**	**lmo2505**	**LMR479a_2630**	**-7.75**	**1.42E-297**
**LM6179_0697**		**NLP/P60 family protein**	**lmo0394**	**LMR479a_0405**	**-7.71**	**4.83E-146**
**LM6179_0987**		**Flagellar biosynthesis protein FliP**	**lmo0676**	**LMR479a_0694**	**-7.38**	**1.13E-62**
LM6179_misc_RNA_26		Purine			-7.33	4.70E-05
**LM6179_0032**	***rplX***	**ribosomal protein L24 (BL23)**	**lmo2621**	**LMR479a_2756**	**-7.28**	**3.15E-193**
**LM6179_0547**	***rplJ***	**ribosomal protein L10 (BL5)**	**lmo0250**	**LMR479a_0262**	**-7.28**	**1.76E-178**
**LM6179_1181**		**conserved membrane protein of unknown function**	**lmo0867**	**LMR479a_0887**	**-7.15**	**2.47E-104**
**LM6179_0030**	***rpsNA***	**ribosomal protein S14**	**lmo2619**	**LMR479a_2754**	**-7.08**	**8.38E-79**
**LM6179_1523**		**N-acetylmuramoyl-L-alanine amidase, family 4**	**lmo1216**	**LMR479a_1237**	**-7.03**	**1.54E-250**
**LM6179_1149**	***ywjA***	**putative ABC lipid transporter (ATP-binding protein)**	**lmo0837**	**LMR479a_0855**	**-6.91**	**4.99E-95**
**LM6179_0698**		**Acetyltransferase**	**lmo0395**	**LMR479a_0406**	**-6.90**	**4.83E-17**
**LM6179_0038**	***rplV***	**ribosomal protein L22 (BL17)**	**lmo2627**	**LMR479a_2762**	**-6.89**	**1.67E-112**
**LM6179_0548**	***rplL***	**ribosomal protein L12 (BL9)**	**lmo0251**	**LMR479a_0263**	**-6.89**	**1.85E-123**
**LM6179_0990**		**Flagellar biosynthesis protein FlhB**	**lmo0679**	**LMR479a_0697**	**-6.88**	**1.09E-45**
**LM6179_0029**	***rpsH***	**ribosomal protein S8 (BS8)**	**lmo2618**	**LMR479a_2753**	**-6.85**	**5.55E-90**
**LM6179_0031**	***rplE***	**ribosomal protein L5 (BL6)**	**lmo2620**	**LMR479a_2755**	**-6.84**	**1.62E-75**
**LM6179_0044**	***rpsJ***	**ribosomal protein S10 (BS13)**	**lmo2633**	**LMR479a_2768**	**-6.83**	**3.10E-75**

**LM6179_0034**	***rpsQ***	**ribosomal protein S17 (BS16)**	**lmo2623**	**LMR479a_2758**	**-6.81**	**8.08E-92**
**LM6179_0027**	***rplR***	**ribosomal protein L18**	**lmo2616**	**LMR479a_2751**	**-6.81**	**3.80E-94**
**LM6179_0039**	***rpsS***	**ribosomal protein S19 (BS19)**	**lmo2628**	**LMR479a_2763**	**-6.80**	**3.88E-143**
**LM6179_0026**	***rpsE***	**ribosomal protein S5**	**lmo2615**	**LMR479a_2750**	**-6.77**	**1.76E-84**
**LM6179_0028**	***rplF***	**ribosomal protein L6 (BL8)**	**lmo2617**	**LMR479a_2752**	**-6.76**	**3.65E-89**
**LM6179_0828**		**conserved membrane protein of unknown function**	**lmo0523**	**LMR479a_0535**	**-6.75**	**2.29E-12**
**LM6179_2821**		**conserved protein of unknown function**	**lmo2050**	**LMR479a_2161**	**-6.75**	**4.12E-140**
**LM6179_0033**	***rplNA***	**ribosomal protein L14**	**lmo2622**	**LMR479a_2757**	**-6.73**	**1.24E-72**
**LM6179_0043**	***rplC***	**ribosomal protein L3 (BL3)**	**lmo2632**	**LMR479a_2767**	**-6.71**	**2.90E-76**
**LM6179_0035**	***rpmC***	**ribosomal protein L29**	**lmo2624**	**LMR479a_2759**	**-6.69**	**1.60E-74**
LM6179_0811		Cell wall surface anchor family protein	lmo0514	LMR479a_0521	-6.69	2.56E-56
**LM6179_3054**	***fruR***	**transcriptional regulator (DeoR family)**	**lmo2337**	**LMR479a_2456**	**-6.68**	**5.78E-53**
**LM6179_0444**	***znuC***	**Zn(II) transporter (ATP-binding protein)**	**lmo0154**	**LMR479a_0163**	**-6.68**	**3.39E-08**
**LM6179_0036**	***rplP***	**ribosomal protein L16**	**lmo2625**	**LMR479a_2760**	**-6.68**	**1.55E-58**
**LM6179_0041**	***rplW***	**ribosomal protein L23**	**lmo2630**	**LMR479a_2765**	**-6.68**	**2.78E-97**
**LM6179_2301**	***folC***	**folyl-polyglutamate synthase**	**lmo1551**	**LMR479a_1644**	**-6.66**	**2.31E-68**
**LM6179_0042**	***rplD***	**ribosomal protein L4**	**lmo2631**	**LMR479a_2766**	**-6.65**	**6.80E-74**
**LM6179_1332**	***opuAB***	**glycine betaine ABC transporter (permease)**	**lmo1015**	**LMR479a_1041**	**-6.62**	**7.13E-127**
**LM6179_0989**		**Flagellar biosynthesis protein FliR**	**lmo0678**	**LMR479a_0696**	**-6.61**	**2.21E-39**
**LM6179_1308**		**Membrane protein**	**lmo0991**	**LMR479a_1017**	**-6.60**	**4.60E-67**
**LM6179_0037**	***rpsC***	**ribosomal protein S3 (BS3)**	**lmo2626**	**LMR479a_2761**	**-6.58**	**4.69E-86**
**LM6179_0040**	***rplB***	**ribosomal protein L2 (BL2)**	**lmo2629**	**LMR479a_2764**	**-6.58**	**5.20E-89**
**LM6179_0025**	***rpmD***	**ribosomal protein L30 (BL27)**	**lmo2614**	**LMR479a_2749**	**-6.50**	**2.96E-66**
**LM6179_2586**	***rpmB***	**ribosomal protein L28**	**lmo1816**	**LMR479a_1926**	**-6.45**	**1.05E-60**

Supplementary Table S6: The 50 most downregulated chromosomal DE genes in the *L. monocytogenes* R479a transcriptome after 30 min exposure to 1% lactic acid. Genes that are part of the Sigma B regulon are highlighted in blue. DE genes which are also downregulated DE genes in 6179 under lactic acid treatment are highlighted in bold.

L. monocytogenes **R479a locus_tag**	**Gene**	**Product**	***L. monocytogenes*** **EGDe locus_tag**	***L. monocytogenes*** **6179 locus_tag**	**log2foldchange**	**Q value**
**LMR479a_2546**		**Cation efflux family protein**	**lmo2423**	**LM6179_1852**	**-10.09**	**2.43E-27**
**LMR479a_2648**	***yocH***	**Cell wall-binding protein yocH**	**lmo2522**	**LM6179_1681**	**-9.97**	**7.91E-157**
LMR479a_2664		Predicted protein (modular protein)			-9.08	1.02E-22
**LMR479a_0195**		**conserved protein of unknown function**	**lmo0186**	**LM6179_0476**	**-8.79**	**2.81E-208**
**LMR479a_2632**	***ftsE***	**cell-division ABC transporter (ATP-binding protein)**	**lmo2507**	**LM6179_1697**	**-8.73**	**1.10E-94**
LMR479a_tRNA4		Glu tRNA			-8.50	2.90E-06
**LMR479a_1040**	***opuAA***	**glycine betaine ABC transporter (ATP-binding protein)**	**lmo1014**	**LM6179_1331**	**-8.40**	**5.00E-72**
**LMR479a_1711**	***ytnP***	**putative metal-dependent hydrolase**	**lmo1614**	**LM6179_2365**	**-8.23**	**2.17E-17**
**LMR479a_2631**	***ftsX***	**cell-division ABC transporter**	**lmo2506**	**LM6179_1698**	**-8.09**	**5.95E-149**
**LMR479a_0262**	***rplJ***	**ribosomal protein L10 (BL5)**	**lmo0250**	**LM6179_0547**	**-7.93**	**3.06E-182**
**LMR479a_1330**		**conserved membrane protein of unknown function**	**lmo1250**	**LM6179_1984**	**-7.89**	**3.23E-11**
**LMR479a_1926**	***rpmB***	**ribosomal protein L28**	**lmo1816**	**LM6179_2586**	**-7.81**	**9.33E-93**
**LMR479a_0406**		**conserved protein of unknown function**	**lmo0395**	**LM6179_0698**	**-7.72**	**3.71E-05**
**LMR479a_0405**		**NLP/P60 family protein**	**lmo0394**	**LM6179_0697**	**-7.66**	**9.06E-80**
**LMR479a_2755**	***rplE***	**ribosomal protein L5 (BL6)**	**lmo2620**	**LM6179_0031**	**-7.64**	**8.02E-81**
**LMR479a_0263**	***rplL***	**ribosomal protein L12 (BL9)**	**lmo0251**	**LM6179_0548**	**-7.60**	**1.16E-146**
**LMR479a_2863**		**conserved protein of unknown function**	**lmo2726**	**LM6179_0140**	**-7.59**	**5.49E-05**
**LMR479a_2765**	***rplW***	**ribosomal protein L23**	**lmo2630**	**LM6179_0041**	**-7.58**	**3.68E-168**
**LMR479a_2768**	***rpsJ***	**ribosomal protein S10 (BS13)**	**lmo2633**	**LM6179_0044**	**-7.56**	**9.89E-62**
**LMR479a_2756**	***rplX***	**ribosomal protein L24 (BL23)**	**lmo2621**	**LM6179_0032**	**-7.51**	**4.22E-100**
**LMR479a_1770**		**conserved exported protein of unknown function**	**lmo1671**	**LM6179_2422**	**-7.50**	**1.21E-29**
**LMR479a_2766**	***rplD***	**ribosomal protein L4**	**lmo2631**	**LM6179_0042**	**-7.44**	**2.29E-122**
**LMR479a_0851**		**conserved protein of unknown function**	**lmo0833**	**LM6179_1145**	**-7.40**	**1.79E-09**
**LMR479a_2763**	***rpsS***	**ribosomal protein S19 (BS19)**	**lmo2628**	**LM6179_0039**	**-7.40**	**3.52E-104**
**LMR479a_2762**	***rplV***	**ribosomal protein L22 (BL17)**	**lmo2627**	**LM6179_0038**	**-7.39**	**9.61E-67**

**LMR479a_2767**	***rplC***	**ribosomal protein L3 (BL3)**	**lmo2632**	**LM6179_0043**	**-7.35**	**8.46E-111**
**LMR479a_2764**	***rplB***	**ribosomal protein L2 (BL2)**	**lmo2629**	**LM6179_0040**	**-7.34**	**1.83E-70**
**LMR479a_0694**		**Flagellar biosynthesis protein FliP**	**lmo0676**	**LM6179_0987**	**-7.33**	**2.60E-14**
**LMR479a_2759**	***rpmC***	**ribosomal protein L29**	**lmo2624**	**LM6179_0035**	**-7.32**	**6.97E-61**
**LMR479a_2751**	***rplR***	**ribosomal protein L18**	**lmo2616**	**LM6179_0027**	**-7.30**	**1.89E-105**
**LMR479a_0887**		**conserved membrane protein of unknown function**	**lmo0867**	**LM6179_1181**	**-7.26**	**9.80E-62**
**LMR479a_1906**	***ylqC***	**putative RNA binding protein**	**lmo1796**	**LM6179_2565**	**-7.25**	**1.59E-108**
**LMR479a_1644**	***folC***	**folyl-polyglutamate synthase**	**lmo1551**	**LM6179_2301**	**-7.25**	**4.02E-27**
**LMR479a_2630**	***spl***	**P45**	**lmo2505**	**LM6179_1699**	**-7.23**	**4.24E-109**
**LMR479a_2752**	***rplF***	**ribosomal protein L6 (BL8)**	**lmo2617**	**LM6179_0028**	**-7.21**	**3.57E-128**
**LMR479a_2754**	***rpsNA***	**ribosomal protein S14**	**lmo2619**	**LM6179_0030**	**-7.21**	**5.08E-59**
**LMR479a_2475**		**conserved protein of unknown function**	**LISIN_2428**	**LM6179_3073**	**-7.19**	**1.87E-04**
**LMR479a_2761**	***rpsC***	**ribosomal protein S3 (BS3)**	**lmo2626**	**LM6179_0037**	**-7.19**	**8.92E-57**
LMR479a_2753	*rpsH*	ribosomal protein S8 (BS8)	lmo2618		-7.13	2.22E-48
**LMR479a_2760**	***rplP***	**ribosomal protein L16**	**lmo2625**	**LM6179_0036**	**-7.12**	**2.63E-44**
**LMR479a_2757**	***rplNA***	**ribosomal protein L14**	**lmo2622**	**LM6179_0033**	**-7.08**	**2.37E-45**
**LMR479a_2750**	***rpsE***	**ribosomal protein S5**	**lmo2615**	**LM6179_0026**	**-7.06**	**7.13E-69**
**LMR479a_1907**	***rpsP***	**ribosomal protein S16 (BS17)**	**lmo1797**	**LM6179_2566**	**-7.06**	**1.48E-98**
**LMR479a_2629**		**conserved exported protein of unknown function**	**lmo2504**	**LM6179_1700**	**-7.05**	**1.86E-74**
**LMR479a_1041**	***opuAB***	**glycine betaine ABC transporter (permease)**	**lmo1015**	**LM6179_1332**	**-6.99**	**3.40E-104**
**LMR479a_2749**	***rpmD***	**ribosomal protein L30 (BL27)**	**lmo2614**	**LM6179_0025**	**-6.97**	**1.02E-68**
**LMR479a_1232**		**putative Membrane protein**	**lmo1211**	**LM6179_1518**	**-6.94**	**2.29E-18**
LMR479a_tRNA38		Thr tRNA			-6.92	2.82E-08
**LMR479a_2989**		**conserved protein of unknown function**	**lmo2852**	**LM6179_0272**	**-6.90**	**2.03E-12**
**LMR479a_2758**	***rpsQ***	**ribosomal protein S17 (BS16)**	**lmo2623**	**LM6179_0034**	**-6.85**	**7.40E-39**

Supplementary Table S7: The 50 most <u>upregulated chromosomal</u> DE genes in the *L. monocytogenes* 6179 transcriptome after 30 min exposure to 1% lactic acid. Genes that are part of the Sigma B regulon are highlighted in blue. DE genes which are also upregulated DE genes in R479a under lactic acid treatment are highlighted in bold.

L. monocytogenes 6179 locus_tag	Gene	Product	*L. monocytogenes* EGDe locus_tag	*L. monocytogenes* R479a locus_tag	log2foldchange	Q value
**LM6179_2993**		**conserved protein of unknown function**	**lmo2213**	**LMR479a_2327**	**9.75**	**4.68E-108**
**LM6179_0569**	***dapE***	**putative succinyl-diaminopimelate desuccinylase**	**lmo0265**	**LMR479a_0284**	**9.65**	**1.56E-211**
**LM6179_3049**		**YhzC protein (fragment)**	**lmo2269**	**LMR479a_2383**	**9.60**	**7.85E-104**
**LM6179_3010**		**putative Arsenate reductase**	**lmo2230**	**LMR479a_2344**	**9.50**	**2.41E-224**
**LM6179_1049**		**conserved protein of unknown function**	**lmo0737**	**LMR479a_0756**	**9.48**	**6.57E-211**
**LM6179_0917**		**protein of unknown function**	**LISIN_0633**	**LMR479a_0623**	**9.26**	**5.59E-25**
**LM6179_1048**	***rpiB***	**ribose 5-phosphate isomerase B/allose 6-phosphate isomerase**	**lmo0736**	**LMR479a_0755**	**9.10**	**3.53E-118**
**LM6179_0743**		**conserved protein of unknown function**	**lmo0439**	**LMR479a_0448**	**9.00**	**3.87E-141**
**LM6179_1047**	***rpe***	**Ribulose-phosphate 3-epimerase**	**lmo0735**	**LMR479a_0754**	**8.98**	**1.04E-136**
**LM6179_1194**		**LysM domain protein (LPXTG motif)**	**lmo0880**	**LMR479a_0901**	**8.97**	**2.56E-177**
**LM6179_2600**		**Short chain dehydrogenase**	**lmo1830**	**LMR479a_1940**	**8.73**	**2.25E-155**
**LM6179_0086**		**conserved protein of unknown function**	**lmo2673**	**LMR479a_2809**	**8.69**	**1.85E-123**
**LM6179_2838**	***cbh***	**Choloylglycine hydrolase**	**lmo2067**	**LMR479a_2178**	**8.52**	**1.10E-150**
**LM6179_misc_ RNA_rli47**	***rli47***	**Rli47**		**R479a_misc_ RNA_rli47**	**8.47**	**6.53E-18**
**LM6179_1050**	***bglP***	**phosphotransferase system (PTS) beta-glucoside-specific enzyme IIBCA component**	**lmo0738**	**LMR479a_0757**	**8.44**	**7.67E-246**
**LM6179_0427**	***yjdI***	**conserved hypothetical protein**	**lmo0133**	**LMR479a_0141**	**8.37**	**1.66E-45**
**LM6179_2618**	***mntC***	**Manganese transport system membrane protein mntC**	**lmo1848**	**LMR479a_1958**	**8.32**	**5.86E-140**
**LM6179_1311**		**conserved membrane protein of unknown function**	**lmo0994**	**LMR479a_1020**	**8.26**	**3.69E-166**
**LM6179_1096**	***manX***	**fragment of fused mannose-specific PTS enzymes: IIA component ; IIB component (part 1)**	**lmo0784**	**LMR479a_0803**	**8.22**	**2.37E-144**
**LM6179_1095**	***manX***	**fragment of fused mannose-specific PTS enzymes: IIA component ; IIB component (part 2)**	**lmo0783**	**LMR479a_0802**	**8.20**	**1.58E-112**
**LM6179_2451**		**conserved protein of unknown function**	**lmo1694**	**LMR479a_1797**	**8.17**	**1.24E-261**
**LM6179_0567**		**Internalin C2**	**lmo0263**	**LMR479a_0281**	**8.16**	**1.11E-164**
**LM6179_0935**		**conserved protein of unknown function**	**lmo0628**	**LMR479a_0641**	**8.14**	**6.76E-54**
**LM6179_1230**		**PTS system protein**	**lmo0914**	**LMR479a_0937**	**8.11**	**1.23E-29**

**LM6179_2617**	***mntA***	**Manganese-binding lipoprotein mntA**	**lmo1847**	**LMR479a_1957**	**8.10**	**3.69E-254**
**LM6179_0916**		**conserved exported protein of unknown function**	**lmo0610**	**LMR479a_0622**	**8.08**	**5.59E-123**
**LM6179_0014**		**conserved membrane protein of unknown function**	**lmo2602**	**LMR479a_2738**	**8.03**	**4.60E-44**
**LM6179_0298**		**conserved exported protein of unknown function**	**lmo0019**	**LMR479a_0019**	**7.99**	**4.75E-88**
**LM6179_0162**	***ydaG***	**General stress protein 26**	**lmo2748**	**LMR479a_2885**	**7.96**	**1.65E-201**
**LM6179_2619**	***mntB***	**manganese ABC transporter (ATP-binding protein)**	**lmo1849**	**LMR479a_1959**	**7.93**	**2.99E-222**
**LM6179_1229**	***gabD***	**succinate-semialdehyde dehydrogenase**	**lmo0913**	**LMR479a_0936**	**7.80**	**2.27E-112**
**LM6179_1975**		**conserved protein of unknown function**	**lmo1241**	**LMR479a_1321**	**7.69**	**0.00E+00**
**LM6179_0428**	***yjdJ***	**putative acyltransferase with acyl-CoA N-acyltransferase domain**	**lmo0134**	**LMR479a_0142**	**7.57**	**1.41E-69**
**LM6179_0980**	***yhxD***	**putative oxidoreductase**	**lmo0669**	**LMR479a_0687**	**7.57**	**1.30E-108**
**LM6179_1626**		**conserved protein of unknown function**	**lmo2573**	**LMR479a_2701**	**7.52**	**3.25E-112**
**LM6179_1231**		**IIC component PTS system**	**lmo0915**	**LMR479a_0938**	**7.52**	**1.02E-74**
**LM6179_1051**	***bglH***	**aryl-phospho-beta-d-glucosidase**	**lmo0739**	**LMR479a_0758**	**7.51**	**2.37E-294**
**LM6179_1880**	***yhfK***	**Uncharacterized sugar epimerase yhfK**	**lmo2391**	**LMR479a_2515**	**7.49**	**3.29E-102**
**LM6179_1107**	***ywnB***	**putative oxidoreductase**	**lmo0794**	**LMR479a_0813**	**7.45**	**1.78E-139**
**LM6179_2857**		**Cell wall surface anchor family protein**	**lmo2085**	**LMR479a_2197**	**7.43**	**1.88E-124**
**LM6179_1033**	***ydaP***	**putative enzyme with pyruvate as substrate**	**lmo0722**	**LMR479a_0740**	**7.36**	**4.96E-110**
**LM6179_1841**		**putative glutamate decarboxylase gamma**	**lmo2434**	**LMR479a_2558**	**7.28**	**5.75E-166**
**LM6179_1884**		**conserved membrane protein of unknown function**	**lmo2387**	**LMR479a_2511**	**7.26**	**1.83E-76**
**LM6179_0123**		**putative PTS system, cellobiose-specific, IIC component**	**lmo2708**	**LMR479a_2846**	**7.26**	**1.14E-81**
**LM6179_1627**		**conserved protein of unknown function**	**lmo2572**	**LMR479a_2700**	**7.23**	**9.26E-78**
**LM6179_1629**		**putative Membrane protein**	**lmo2570**	**LMR479a_2698**	**7.16**	**8.83E-105**
**LM6179_0901**		**conserved membrane protein of unknown function**	**lmo0596**	**LMR479a_0608**	**7.12**	**3.06E-161**
**LM6179_1252**		**conserved protein of unknown function**	**lmo0937**	**LMR479a_0959**	**7.08**	**4.50E-43**
**LM6179_0734**		**Internalin-A (fragment)**	**lmo0433**	**LMR479a_0443**	**7.07**	**1.31E-92**
**LM6179_1232**		**conserved protein of unknown function**	**lmo0916**	**LMR479a_0939**	**7.05**	**3.13E-41**

Supplementary Table S8: The 50 most <u>upregulated</u> chromosomal DE genes in the *L. monocytogenes* R479a transcriptome after 30 min exposure to 1% lactic acid. Genes that are part of the Sigma B regulon are highlighted in blue. DE genes which are also upregulated DE genes in 6179 under hydrogen peroxide treatment are highlighted in bold.

L. monocytogenes R479a locus_tag	Gene	Product	*L. monocytogenes* EGDe locus_tag	*L. monocytogenes* 6179 locus_tag	log2fold change	Q value
**LMR479a_0356**	***rpiB***	**Ribose-5-phosphate isomerase B**	**lmo0345**	**LM6179_0645**	**10.13**	**4.87E-94**
**LMR479a_0284**	***dapE***	**putative succinyl-diaminopimelate desuccinylase**		**LM6179_0569**	**10.00**	**4.43E-174**
**R479a_misc_ RNA_rli47**	***rli47***	**Rli47**		**LM6179_misc_ RNA_rli47**	**9.97**	**1.88E-86**
**LMR479a_0360**		**conserved membrane protein of unknown function**	**lmo0349**	**LM6179_0649**	**9.97**	**2.78E-49**
**LMR479a_1957**	***mntA***	**Manganese-binding lipoprotein mntA**	**lmo1847**	**LM6179_2617**	**9.89**	**1.22E-197**
**LMR479a_2383**		**YhzC protein (fragment)**	**lmo2269**	**LM6179_3049**	**9.89**	**4.51E-113**
**LMR479a_2344**		**conserved protein of unknown function**	**lmo2230**	**LM6179_3010**	**9.85**	**0**
**LMR479a_0641**		**conserved protein of unknown function**	**lmo0628**	**LM6179_0935**	**9.75**	**1.23E-40**
**LMR479a_1958**	***mntC***	**Manganese transport system membrane protein mntC**	**lmo1848**	**LM6179_2618**	**9.70**	**5.85E-254**
**LMR479a_0361**		**conserved membrane protein of unknown function**	**lmo0350**	**LM6179_0650**	**9.59**	**1.35E-46**
**LMR479a_2327**		**conserved protein of unknown function**	**lmo2213**	**LM6179_2993**	**9.57**	**1.73E-141**
**LMR479a_2715**	***yrhE***	**putative oxido-reductase**	**lmo2586**	**LM6179_1612**	**9.54**	**6.78E-261**
**LMR479a_2809**		**conserved protein of unknown function**	**lmo2673**	**LM6179_0086**	**9.37**	**1.52E-234**
**LMR479a_0900**		**conserved exported protein of unknown function**	**lmo0880**	**LM6179_1194**	**9.26**	**2.95E-106**
**LMR479a_0359**		**conserved protein of unknown function**	**lmo0348**	**LM6179_0648**	**9.11**	**3.80E-25**
**LMR479a_0281**	***inlC2***	**Internalin C2**	**lmo0263**	**LM6179_0567**	**9.04**	**9.83E-255**
**LMR479a_0355**		**Short chain dehydrogenase**	**lmo0344**	**LM6179_0644**	**8.90**	**4.08E-100**
**LMR479a_2178**	***cbh***	**Choloylglycine hydrolase**	**lmo2067**	**LM6179_2838**	**8.89**	**1.81E-135**
**LMR479a_0901**		**conserved protein of unknown function**	**lmo0880**	**LM6179_1194**	**8.73**	**1.58E-179**
**LMR479a_0354**	***ywjH***	**putative transaldolase**	**lmo0343**	**LM6179_0643**	**8.65**	**1.04E-68**
**LMR479a_0358**	***dhaL***	**PTS-dependent dihydroxyacetone kinase, ADP-binding subunit dhaL**	**lmo0347**	**LM6179_0647**	**8.60**	**1.69E-140**
**LMR479a_0622**		**conserved exported protein of unknown function**	**lmo0610**	**LM6179_0916**	**8.48**	**7.21E-267**
**LMR479a_0663**		**conserved membrane protein of unknown function**	**lmo0647**	**LM6179_0957**	**8.44**	**6.12E-61**
**LMR479a_2917**	***gmuA***	**oligo-alpha-mannoside phosphotransferase system enzyme IIA**	**lmo2780**	**LM6179_0201**	**8.43**	**3.75E-116**
**LMR479a_0443**	***inlA***	**Internalin-A**	**lmo0433**	**LM6179_0734**	**8.43**	**4.16E-235**

**LMR479a_0937**		**conserved exported protein of unknown function**	**lmo0914**	**LM6179_1230**	**8.38**	**6.69E-30**
**LMR479a_2713**	***fdhD***	**Protein fdhD homolog**	**lmo2584**	**LM6179_1614**	**8.38**	**3.24E-89**
**LMR479a_0623**		**protein of unknown function**	**LISIN_0633**	**LM6179_0917**	**8.37**	**2.99E-29**
**LMR479a_2714**		**conserved protein of unknown function**	**lmo2585**	**LM6179_1613**	**8.25**	**1.90E-124**
**LMR479a_0336**		**conserved exported protein of unknown function**	**lmo0324**	**LM6179_0623**	**8.24**	**1.75E-23**
**LMR479a_2846**		**conserved membrane protein of unknown function**	**lmo2708**	**LM6179_0123**	**8.23**	**1.43E-122**
**LMR479a_2822**	***licA***	**phosphotransferase system (PTS) lichenan-specific enzyme IIA component**	**lmo2685**	**LM6179_0099**	**8.21**	**1.01E-112**
**LMR479a_0938**		**PTS system protein**	**lmo0915**	**LM6179_1231**	**8.17**	**1.38E-84**
**LMR479a_2579**		**conserved protein of unknown function**	**lmo2454**	**LM6179_1818**	**8.16**	**5.28E-243**
**LMR479a_2558**		**putative glutamate decarboxylase gamma**	**lmo2434**	**LM6179_1841**	**8.08**	**3.84E-154**
**LMR479a_2197**		**Peptidoglycan binding protein**	**lmo2085**	**LM6179_2857**	**8.00**	**8.01E-170**
**LMR479a_2701**		**conserved protein of unknown function**	**lmo2573**	**LM6179_1626**	**8.00**	**1.67E-108**
**LMR479a_0939**		**conserved protein of unknown function**	**lmo0916**	**LM6179_1232**	**8.00**	**5.46E-53**
**LMR479a_2738**		**conserved membrane protein of unknown function**	**lmo2602**	**LM6179_0014**	**7.94**	**1.57E-77**
**LMR479a_2918**		**conserved protein of unknown function**	**lmo2781**	**LM6179_0202**	**7.93**	**3.42E-241**
**LMR479a_2515**	***yhfK***	**Uncharacterized sugar epimerase yhfK**	**lmo2391**	**LM6179_1880**	**7.93**	**2.46E-130**
**LMR479a_2700**		**conserved protein of unknown function**	**lmo2572**	**LM6179_1627**	**7.79**	**2.74E-104**
**LMR479a_2821**	***licC***	**phosphotransferase system (PTS) lichenan-specific enzyme IIC component**	**lmo2684**	**LM6179_0098**	**7.76**	**5.07E-176**
**LMR479a_2919**		**PTS system protein**	**lmo2782**	**LM6179_0203**	**7.75**	**2.46E-98**
**LMR479a_1020**		**conserved membrane protein of unknown function**	**lmo0994**	**LM6179_1311**	**7.71**	**1.69E-87**
**LMR479a_0141**		**conserved protein of unknown function**	**lmo0133**	**LM6179_0427**	**7.70**	**6.33E-36**
**LMR479a_2885**	***ydaG***	**General stress protein 26**	**lmo2748**	**LM6179_0162**	**7.59**	**1.32E-106**
**LMR479a_1321**		**conserved protein of unknown function**	**lmo1241**	**LM6179_1975**	**7.52**	**8.19E-164**
**LMR479a_0353**	***tkt***	**transketolase**	**lmo0342**	**LM6179_0642**	**7.48**	**3.22E-62**
LMR479a_0282	*inlD*	Internalin D			7.48	5.50E-191

Supplementary Table 9: Genes used to compose the σ^B panel. Genes in the table are composed of all genes from Raengpradub et al. predicted to have σ^B promoters based on a Hidden Markov model, the genes upregulated by σ^B based on RNA-Seq conducted by Oliver et al., and all members of the σ^B regulon with EGD-e locus tags found via literature review conducted by Liu et al. An "X" denotes that the gene was found to be σ^B-dependent in the given study.

	**Raengpradub et al., 2008 (*Appl Environ Microbiol*) – genes with σ^B-dependent promoters based on Hidden Markov model**	**Oliver et al., 2009 (*BMC Genomics*) – genes upregulated by σ^B based on RNA-Seq of stationary phase cells**	**Liu et al., 2019 (*Future Microbiol*) – genes identified in literature review with EGD-e locus tags**
**lmo0007**	X		
**lmo0013**	X		X
**lmo0014**			X
**lmo0015**			X
**lmo0016**			X
**lmo0019**		X	X
**lmo0036**			X
**lmo0037**	X		X
**lmo0043**		X	X
**lmo0052**	X		
**lmo0060**	X		
**lmo0071**	X		
**lmo0075**			X
**lmo0076**			X
**lmo0079**	X		
**lmo0080**	X		
**lmo0091**	X		
**lmo0095**	X		
**lmo0105**			X
**lmo0122**		X	
**lmo0133**	X	X	X
**lmo0134**		X	X
**lmo0169**		X	X
**lmo0170**		X	X
**lmo0186**	X		
**lmo0200**	X		X
**lmo0210**	X		X
**lmo0211**	X		
**lmo0220**	X		
**lmo0221**			X
**lmo0222**			X
**lmo0228**	X		
**lmo0230**	X		X

lmo0231			X
lmo0232			X
lmo0258	X		
lmo0263	X	X	X
lmo0265	X	X	X
lmo0274	X	X	X
lmo0288	X		
lmo0292			X
lmo0305	X		
lmo0315	X		
lmo0321	X	X	X
lmo0325	X		
lmo0334	X		
lmo0336	X		X
lmo0337	X		
lmo0338	X		
lmo0352	X		
lmo0358	X		
lmo0359			X
lmo0360			X
lmo0372		X	
lmo0373			X
lmo0380	X		
lmo0383	X		
lmo0398			X
lmo0399			X
lmo0400			X
lmo0401			X
lmo0402			X
lmo0405	X	X	X
lmo0406			X
lmo0407			X
lmo0408			X
lmo0415	X		
lmo0433		X	X
lmo0434		X	X
lmo0438	X		
lmo0439	X	X	X
lmo0445		X	
lmo0450	X		
lmo0460			X

**lmo0461**			**X**
**lmo0462**			**X**
**lmo0463**			**X**
**lmo0464**			**X**
**lmo0472**	**X**		
**lmo0485**	**X**		
**lmo0489**	**X**		
**lmo0496**	**X**		
**lmo0515**		**X**	**X**
**lmo0523**	**X**		
**lmo0524**	**X**		**X**
**lmo0527**	**X**		**X**
**lmo0529**			**X**
**lmo0530**			**X**
**lmo0539**	**X**	**X**	**X**
**lmo0551**			**X**
**lmo0554**	**X**	**X**	**X**
**lmo0555**		**X**	**X**
**lmo0579**	**X**		**X**
**lmo0580**			**X**
**lmo0582**	**X**		
**lmo0584**	**X**		
**lmo0589**			**X**
**lmo0590**			**X**
**lmo0591**	**X**		**X**
**lmo0592**			**X**
**lmo0593**	**X**	**X**	**X**
**lmo0596**	**X**	**X**	**X**
**lmo0602**		**X**	**X**
**lmo0605**	**X**		
**lmo0610**	**X**	**X**	**X**
**lmo0623**	**X**		
**lmo0628**		**X**	**X**
**lmo0629**	**X**	**X**	**X**
**lmo0641**	**X**		
**lmo0643**	**X**		
**lmo0647**			**X**
**lmo0648**			**X**
**lmo0654**	**X**	**X**	**X**
**lmo0655**		**X**	**X**
**lmo0669**	**X**	**X**	**X**

**lmo0670**		**X**	**X**
**lmo0671**			**X**
**lmo0722**	**X**	**X**	**X**
**lmo0723**			**X**
**lmo0724**			**X**
**lmo0759**	**X**		
**lmo0781**		**X**	**X**
**lmo0782**		**X**	**X**
**lmo0783**		**X**	**X**
**lmo0784**	**X**	**X**	**X**
**lmo0794**	**X**	**X**	**X**
**lmo0796**		**X**	**X**
**lmo0805**	**X**		
**lmo0808**	**X**		
**lmo0813**	**X**		
**lmo0819**		**X**	
**lmo0821**			**X**
**lmo0830**	**X**		
**lmo0838**	**X**		
**lmo0839**	**X**		
**lmo0850**	**X**		
**lmo0863**	**X**		
**lmo0869**			**X**
**lmo0870**	**X**		**X**
**lmo0880**	**X**	**X**	**X**
**lmo0893**	**X**		**X**
**lmo0894**	**X**		**X**
**lmo0895**			**X**
**lmo0896**	**X**		**X**
**lmo0911**	**X**	**X**	**X**
**lmo0913**		**X**	**X**
**lmo0929**			**X**
**lmo0937**	**X**	**X**	**X**
**lmo0944**	**X**		
**lmo0953**	**X**	**X**	**X**
**lmo0956**	**X**		**X**
**lmo0957**			**X**
**lmo0958**			**X**
**lmo0993**	**X**		
**lmo0994**	**X**	**X**	**X**
**lmo0995**			**X**

lmo1005	X		
lmo1014			X
lmo1015			X
lmo1016			X
lmo1018	X		
lmo1029	X		
lmo1052	X		
lmo1064	X		
lmo1067			X
lmo1068	X		X
lmo1072	X		
lmo1096	X		
lmo1123	X		
lmo1124	X		
lmo1140		X	X
lmo1149	X		
lmo1151			X
lmo1152			X
lmo1153			X
lmo1154			X
lmo1156			X
lmo1159			X
lmo1164			X
lmo1168			X
lmo1226			X
lmo1241		X	X
lmo1258	X		
lmo1261	X		X
lmo1264	X		
lmo1295	X	X	X
lmo1299	X		
lmo1300	X		X
lmo1319	X		
lmo1337	X		
lmo1339			X
lmo1340	X		X
lmo1367			X
lmo1369	X		
lmo1370	X		
lmo1375	X	X	X
lmo1378	X		

lmo1394	X		
lmo1403	X		
lmo1421	X	X	X
lmo1422			X
lmo1425		X	X
lmo1426		X	X
lmo1427			X
lmo1428	X		X
lmo1432			X
lmo1433	X	X	X
lmo1439	X		
lmo1451	X		
lmo1454	X		X
lmo1459	X		
lmo1487	X		
lmo1497	X		
lmo1517	X		
lmo1518	X		
lmo1526	X	X	X
lmo1528	X		
lmo1534	X		
lmo1538			X
lmo1539	X		X
lmo1565	X		
lmo1570	X		
lmo1571			X
lmo1580	X		X
lmo1583	X		
lmo1597	X		
lmo1601			X
lmo1602	X	X	X
lmo1604	X		
lmo1605			X
lmo1606	X	X	X
lmo1650	X		
lmo1652	X		
lmo1655	X		
lmo1666	X		X
lmo1674	X		
lmo1688	X		
lmo1690	X		

**lmo1694**	**X**	**X**	**X**
**lmo1698**	**X**	**X**	**X**
**lmo1701**			**X**
**lmo1702**			**X**
**lmo1703**			**X**
**lmo1704**	**X**		**X**
**lmo1713**	**X**		**X**
**lmo1743**	**X**		
**lmo1783**	**X**		
**lmo1789**			**X**
**lmo1790**			**X**
**lmo1816**	**X**		
**lmo1830**	**X**	**X**	**X**
**lmo1831**			**X**
**lmo1832**			**X**
**lmo1833**			**X**
**lmo1834**			**X**
**lmo1835**			**X**
**lmo1836**	**X**		**X**
**lmo1837**			**X**
**lmo1838**			**X**
**lmo1839**	**X**		
**lmo1855**	**X**		
**lmo1861**	**X**		
**lmo1866**		**X**	
**lmo1883**	**X**	**X**	**X**
**lmo1902**	**X**		
**lmo1920**	**X**		
**lmo1929**			**X**
**lmo1930**			**X**
**lmo1931**			**X**
**lmo1932**			**X**
**lmo1933**	**X**		**X**
**lmo1958**	**X**		
**lmo1992**	**X**		**X**
**lmo1993**	**X**		
**lmo2003**		**X**	
**lmo2042**	**X**		
**lmo2048**	**X**		
**lmo2066**	**X**		
**lmo2067**	**X**	**X**	**X**

lmo2080	X		
lmo2085	X	X	X
lmo2092	X		X
lmo2093	X		
lmo2094			X
lmo2095			X
lmo2122	X		
lmo2130		X	X
lmo2132		X	X
lmo2153	X		
lmo2156	X		X
lmo2157	X	X	X
lmo2158	X	X	X
lmo2172	X		
lmo2173	X		
lmo2174			X
lmo2175			X
lmo2190			X
lmo2191	X		X
lmo2203	X		
lmo2205	X		X
lmo2210	X		
lmo2213	X	X	X
lmo2215	X		
lmo2216	X		
lmo2230	X	X	X
lmo2231		X	X
lmo2232			X
lmo2236	X		
lmo2269		X	X
lmo2272	X		
lmo2287	X		
lmo2330	X		
lmo2358	X		X
lmo2362			X
lmo2363			X
lmo2386	X		X
lmo2387	X	X	X
lmo2389	X		
lmo2391	X	X	X
lmo2398	X	X	X

lmo2399	X		
lmo2434	X	X	X
lmo2438	X		
lmo2454		X	X
lmo2455			X
lmo2456			X
lmo2457			X
lmo2458			X
lmo2459			X
lmo2460	X		X
lmo2462			X
lmo2463	X	X	X
lmo2467	X		
lmo2468			X
lmo2472	X		
lmo2477			X
lmo2484		X	X
lmo2485	X	X	X
lmo2486	X		
lmo2494		X	X
lmo2511	X		X
lmo2556	X		
lmo2570	X	X	X
lmo2571		X	X
lmo2572		X	X
lmo2573	X	X	X
lmo2576	X		
lmo2578	X		
lmo2589	X		
lmo2602	X	X	X
lmo2603		X	X
lmo2638	X		
lmo2640	X		
lmo2642	X		
lmo2665			X
lmo2666			X
lmo2667			X
lmo2668			X
lmo2670	X	X	X
lmo2671		X	X
lmo2672		X	X

**lmo2673**	**X**	**X**	**X**
**lmo2674**		**X**	**X**
**lmo2684**	**X**		
**lmo2695**	**X**		**X**
**lmo2696**			**X**
**lmo2697**			**X**
**lmo2704**	**X**		
**lmo2724**	**X**	**X**	**X**
**lmo2733**		**X**	**X**
**lmo2735**			**X**
**lmo2741**	**X**		
**lmo2746**			**X**
**lmo2747**			**X**
**lmo2748**		**X**	**X**
**lmo2776**	**X**		
**lmo2783**	**X**		
**lmo2788**	**X**		
**lmo2809**	**X**		
**lmo2823**	**X**		
**lmo2830**	**X**		
**lmo2834**			**X**
**lmo2835**			**X**
**lmo2836**			**X**
**lmo2837**			**X**
**lmo2846**	**X**		
**rli47**		**X**	**X**

Supplementary Table S10: The 50 most upregulated chromosomal DE genes encoding conserved hypothetical proteins in the *L. monocytogenes* 6179 transcriptome after 30 min exposure to 1% lactic acid. Genes that are part of the Sigma B regulon are highlighted in blue. DE genes which are also upregulated DE genes in R479a under lactic acid treatment are highlighted in bold. The putative arsenate reductase (lmo2230), although annotated as a conserved protein of unknown function, is not included in this table.

L. monocytogenes 6179 locus_tag	Product	*L. monocytogenes* EGDe locus_tag	*L. monocytogenes* R479a locus_tag	log2foldchange	Q value
**LM6179_0916**	**conserved protein of unknown function**	**lmo2213**	**LMR479a_2327**	**9.75**	**4.68E-108**
**LM6179_0298**	**conserved protein of unknown function**	**lmo0737**	**LMR479a_0756**	**9.48**	**6.57E-211**
**LM6179_2417**	**conserved protein of unknown function**	**lmo0439**	**LMR479a_0448**	**9.00**	**3.87E-141**
**LM6179_2352**	**conserved protein of unknown function**	**lmo2673**	**LMR479a_2809**	**8.69**	**1.85E-123**
**LM6179_0630**	**conserved hypothetical protein**	**lmo0133**	**LMR479a_0141**	**8.37**	**1.66E-45**
**LM6179_0623**	**conserved membrane protein of unknown function**	**lmo0994**	**LMR479a_1020**	**8.26**	**3.69E-166**
**LM6179_1388**	**conserved protein of unknown function**	**lmo1694**	**LMR479a_1797**	**8.17**	**1.24E-261**
**LM6179_1034**	**conserved protein of unknown function**	**lmo0628**	**LMR479a_0641**	**8.14**	**6.76E-54**
**LM6179_0804**	**conserved exported protein of unknown function**	**lmo0610**	**LMR479a_0622**	**8.08**	**5.59E-123**
**LM6179_1631**	**conserved membrane protein of unknown function**	**lmo2602**	**LMR479a_2738**	**8.03**	**4.60E-44**
**LM6179_0780**	**conserved exported protein of unknown function**	**lmo0019**	**LMR479a_0019**	**7.99**	**4.75E-88**
**LM6179_1269**	**conserved protein of unknown function**	**lmo1241**	**LMR479a_1321**	**7.69**	**0.00E+00**
**LM6179_1968**	**conserved protein of unknown function**	**lmo2573**	**LMR479a_2701**	**7.52**	**3.25E-112**
**LM6179_1621**	**conserved membrane protein of unknown function**	**lmo2387**	**LMR479a_2511**	**7.26**	**1.83E-76**
**LM6179_0121**	**conserved protein of unknown function**	**lmo2572**	**LMR479a_2700**	**7.23**	**9.26E-78**
**LM6179_2570**	**conserved membrane protein of unknown function**	**lmo0596**	**LMR479a_0608**	**7.12**	**3.06E-161**
**LM6179_2083**	**conserved protein of unknown function**	**lmo0937**	**LMR479a_0959**	**7.08**	**4.50E-43**
**LM6179_0711**	**conserved protein of unknown function**	**lmo0916**	**LMR479a_0939**	**7.05**	**3.13E-41**
**LM6179_2997**	**conserved protein of unknown function**	**lmo2571**	**LMR479a_2699**	**7.03**	**3.55E-77**
**LM6179_0326**	**conserved protein of unknown function**	**lmo0670**	**LMR479a_0688**	**6.94**	**6.99E-81**
**LM6179_2360**	**conserved protein of unknown function**	**lmo2585**	**LMR479a_2714**	**6.93**	**2.08E-169**
**LM6179_1359**	**conserved protein of unknown function**	**lmo0944**	**LMR479a_0966**	**6.46**	**1.90E-185**
**LM6179_1431**	**conserved protein of unknown function**	**lmo0025**	**LMR479a_0025**	**6.41**	**4.95E-32**
**LM6179_2956**	**conserved protein of unknown function**	**lmo0602**	**LMR479a_0614**	**6.35**	**7.15E-121**
**LM6179_2027**	**conserved membrane protein of unknown function**	**lmo0321**	**LMR479a_0333**	**6.29**	**4.19E-71**

**LM6179_1195**	**conserved protein of unknown function**	**lmo2454**	**LMR479a_2579**	**6.29**	**8.46E-297**
**LM6179_1436**	**conserved protein of unknown function**	**lmo2432**	**LMR479a_2556**	**6.19**	**6.71E-127**
**LM6179_1265**	**conserved membrane protein of unknown function**	**lmo0647**	**LMR479a_0663**	**6.10**	**7.23E-39**
**LM6179_2632**	**conserved protein of unknown function**	**lmo0654**	**LMR479a_0671**	**5.83**	**5.24E-163**
**LM6179_0982**	**conserved protein of unknown function**	**lmo2132**	**LMR479a_2244**	**5.72**	**5.17E-99**
**LM6179_1104**	**conserved protein of unknown function**	**lmo1256**	**LMR479a_1339**	**5.61**	**1.66E-47**
**LM6179_2894**	**conserved protein of unknown function**	**lmo2158**	**LMR479a_2270**	**5.51**	**7.01E-68**
**LM6179_0835**	**conserved membrane protein of unknown function**	**lmo2463**	**LMR479a_2588**	**5.45**	**1.23E-47**
**LM6179_2257**	**conserved membrane protein of unknown function**	**lmo0349**	**LMR479a_0360**	**5.41**	**1.65E-07**
**LM6179_1859**	**conserved protein of unknown function**	**lmo2603**	**LMR479a_2739**	**5.40**	**1.45E-44**
**LM6179_0050**	**conserved protein of unknown function**	**lmo1140**	**LMR479a_1161**	**5.31**	**1.11E-93**
**LM6179_0924**	**conserved protein of unknown function**	**lmo0109**	**LMR479a_0117**	**5.24**	**9.26E-45**
LM6179_2672	conserved protein of unknown function	lmo2742	LMR479a_2879	5.13	5.47E-118
**LM6179_1685**	**conserved protein of unknown function**	**lmo1580**	**LMR479a_1674**	**5.11**	**1.14E-76**
LM6179_2852	conserved exported protein of unknown function	lmo1666	LMR479a_1765	5.09	1.29E-65
**LM6179_2797**	**conserved protein of unknown function**	**lmo0911**	**LMR479a_0934**	**5.08**	**5.95E-69**
LM6179_2682	conserved membrane protein of unknown function	lmo2733	LMR479a_2870	5.07	8.64E-45
**LM6179_0048**	**conserved protein of unknown function**	**lmo2798**	**LMR479a_2935**	**5.03**	**1.66E-23**
**LM6179_2926**	**conserved protein of unknown function**	**lmo0323**	**LMR479a_0335**	**4.80**	**2.05E-88**
LM6179_0906	conserved protein of unknown function	lmo2797	LMR479a_2934	4.77	1.42E-12
LM6179_2851	conserved exported protein of unknown function	lmo1602	LMR479a_1698	4.75	4.07E-74
**LM6179_0232**	**conserved protein of unknown function**	**lmo1601**	**LMR479a_1697**	**4.73**	**2.08E-70**
**LM6179_2512**	**conserved protein of unknown function**	**lmo0357**	**LMR479a_0368**	**4.64**	**8.67E-08**
**LM6179_2400**	**conserved protein of unknown function**	**lmo0515**	**LMR479a_0522**	**4.60**	**1.63E-39**
**LM6179_2834**	**conserved protein of unknown function**	**lmo2671**	**LMR479a_2807**	**4.58**	**1.69E-21**

Supplementary Table S11: The 50 most upregulated chromosomal DE genes encoding conserved hypothetical proteins in the *L. monocytogenes* R479a transcriptome after 30 min exposure to 1% lactic acid. Genes that are part of the Sigma B regulon are highlighted in blue. DE genes which are also upregulated DE genes in 6179 under hydrogen peroxide treatment are highlighted in bold. The putative arsenate reductase (lmo2230), although annotated in R479a as a conserved protein of unknown function, is not included in this table.

***L. monocytogenes* R479a locus_tag**	**Product**	***L. monocytogenes* EGDe locus_tag**	***L. monocytogenes* 6179 locus_tag**	**log2fold change**	**Q value**
**LMR479a_0360**	**conserved membrane protein of unknown function**	**lmo0349**	**LM6179_0649**	**9.97**	**2.78E-49**
**LMR479a_0641**	**conserved protein of unknown function**	**lmo0628**	**LM6179_0935**	**9.75**	**1.23E-40**
**LMR479a_0361**	**conserved membrane protein of unknown function**	**lmo0350**	**LM6179_0650**	**9.59**	**1.35E-46**
**LMR479a_2327**	**conserved protein of unknown function**	**lmo2213**	**LM6179_2993**	**9.57**	**1.73E-141**
**LMR479a_2809**	**conserved protein of unknown function**	**lmo2673**	**LM6179_0086**	**9.37**	**1.52E-234**
LMR479a_0900	conserved exported protein of unknown function	lmo0880	LM6179_1194	9.26	2.95E-106
LMR479a_0359	conserved protein of unknown function	lmo0348	LM6179_0648	9.11	3.80E-25
LMR479a_0901	conserved protein of unknown function	lmo0880	LM6179_1194	8.73	1.58E-179
**LMR479a_0622**	**conserved exported protein of unknown function**	**lmo0610**	**LM6179_0916**	**8.48**	**7.21E-267**
**LMR479a_0663**	**conserved membrane protein of unknown function**	**lmo0647**	**LM6179_0957**	**8.44**	**6.12E-61**
LMR479a_0937	conserved exported protein of unknown function	lmo0914	LM6179_1230	8.38	6.69E-30
**LMR479a_2714**	**conserved protein of unknown function**	**lmo2585**	**LM6179_1613**	**8.25**	**1.90E-124**
**LMR479a_0336**	**conserved exported protein of unknown function**	**lmo0324**	**LM6179_0623**	**8.24**	**1.75E-23**
LMR479a_2846	conserved membrane protein of unknown function	lmo2708	LM6179_0123	8.23	1.43E-122
**LMR479a_2579**	**conserved protein of unknown function**	**lmo2454**	**LM6179_1818**	**8.16**	**5.28E-243**
**LMR479a_2701**	**conserved protein of unknown function**	**lmo2573**	**LM6179_1626**	**8.00**	**1.67E-108**
**LMR479a_0939**	**conserved protein of unknown function**	**lmo0916**	**LM6179_1232**	**8.00**	**5.46E-53**
**LMR479a_2738**	**conserved membrane protein of unknown function**	**lmo2602**	**LM6179_0014**	**7.94**	**1.57E-77**
**LMR479a_2918**	**conserved protein of unknown function**	**lmo2781**	**LM6179_0202**	**7.93**	**3.42E-241**
**LMR479a_2700**	**conserved protein of unknown function**	**lmo2572**	**LM6179_1627**	**7.79**	**2.74E-104**
**LMR479a_1020**	**conserved membrane protein of unknown function**	**lmo0994**	**LM6179_1311**	**7.71**	**1.69E-87**
**LMR479a_0141**	**conserved protein of unknown function**	**lmo0133**	**LM6179_0427**	**7.70**	**6.33E-36**
**LMR479a_1321**	**conserved protein of unknown function**	**lmo1241**	**LM6179_1975**	**7.52**	**8.19E-164**
**LMR479a_2699**	**conserved protein of unknown function**	**lmo2571**	**LM6179_1628**	**7.25**	**8.02E-81**
LMR479a_1834	conserved exported protein of unknown function	lmo1730	LM6179_2489	7.18	3.36E-144

**LMR479a_1797**	**conserved protein of unknown function**	**lmo1694**	**LM6179_2451**	**7.07**	**2.09E-114**
**LMR479a_0362**	**conserved protein of unknown function**	**lmo0351**	**LM6179_0651**	**7.07**	**4.92E-21**
LMR479a_2920	conserved membrane protein of unknown function	lmo2783	LM6179_0204	6.85	1.38E-93
**LMR479a_2556**	**conserved protein of unknown function**	**lmo2432**	**LM6179_1843**	**6.84**	**1.57E-159**
**LMR479a_0688**	**conserved protein of unknown function**	**lmo0670**	**LM6179_0981**	**6.66**	**6.34E-62**
**LMR479a_0522**	**conserved protein of unknown function**	**lmo0515**	**LM6179_0813**	**6.58**	**1.17E-199**
LMR479a_2206	conserved protein of unknown function	lmo2095	LM6179_2866	6.45	2.37E-10
LMR479a_0977	conserved exported protein of unknown function	lmo0953	LM6179_1268	6.41	9.67E-97
**LMR479a_0448**	**conserved protein of unknown function**	**lmo0439**	**LM6179_0743**	**6.34**	**5.73E-137**
**LMR479a_2511**	**conserved membrane protein of unknown function**	**lmo2387**	**LM6179_1884**	**6.27**	**5.23E-108**
**LMR479a_2739**	**conserved protein of unknown function**	**lmo2603**	**LM6179_0015**	**6.17**	**1.45E-102**
LMR479a_0142	conserved protein of unknown function	lmo0134	LM6179_0428	6.17	1.56E-68
**LMR479a_0025**	**conserved protein of unknown function**	**lmo0025**	**LM6179_0304**	**6.15**	**1.10E-28**
**LMR479a_1674**	**conserved protein of unknown function**	**lmo1580**	**LM6179_2331**	**6.14**	**4.31E-137**
LMR479a_0473	conserved protein of unknown function	#N/A	#N/A	6.07	1.04E-16
**LMR479a_0333**	**conserved membrane protein of unknown function**	**lmo0321**	**LM6179_0620**	**6.04**	**9.40E-79**
LMR479a_2800	conserved protein of unknown function	lmo2664	LM6179_0077	5.95	4.52E-51
LMR479a_0352	conserved protein of unknown function	lmo0341	LM6179_0641	5.93	2.93E-67
**LMR479a_0293**	**conserved protein of unknown function**	**lmo0274**	**LM6179_0579**	**5.92**	**2.78E-101**
**LMR479a_1096**	**conserved exported protein of unknown function**	**lmo1068**	**LM6179_1388**	**5.87**	**2.74E-45**
**LMR479a_0934**	**conserved protein of unknown function**	**lmo0911**	**LM6179_1227**	**5.79**	**2.09E-90**
LMR479a_1992	conserved exported protein of unknown function	lmo1883	LM6179_2652	5.79	3.67E-77
**LMR479a_0614**	**conserved protein of unknown function**	**lmo0602**	**LM6179_0907**	**5.72**	**4.71E-87**
**LMR479a_2588**	**conserved membrane protein of unknown function**	**lmo2463**	**LM6179_1809**	**5.68**	**5.76E-75**
**LMR479a_0019**	**conserved exported protein of unknown function**	**lmo0019**	**LM6179_0298**	**5.67**	**1.16E-77**

Supplementary Table S12: Differentially expressed genes on pLM6179 after 30 min exposure to 1% lactic acid. Genes that are also present on pLMR479a are highlighted in grey.

DE genes, putative function	Locus_tag	Log2fold change	Q value	DE gene in pLMR479a 1% lactic acid, pLMR479a locus_tag
Heat shock protein *clpL* – stress response	LM6179_RS15400	3.15	2.50E-29	
conserved exported protein of unknown function	LM6179_RS15220	2.73	1.83E-03	
conserved protein of unknown function	LM6179_RS15375	2.20	7.68E-23	
parA family protein	LM6179_RS15370	2.10	6.35E-15	
conserved membrane protein of unknown function	LM6179_RS15245	2.05	1.48E-05	
conserved protein of unknown function	LM6179_RS15205	1.76	5.36E-03	
conserved protein of unknown function	LM6179_RS15195	1.60	1.83E-03	
tnpA - transposase Tn*5422*	LM6179_RS15360	1.54	3.02E-16	
tnpR - transposase Tn*5422*	LM6179_RS15355	1.41	3.00E-11	
conserved protein of unknown function	LM6179_RS15200	1.40	8.99E-03	
conserved protein of unknown function	LM6179_RS15215	1.38	3.15E-03	
conserved protein of unknown function	LM6179_RS15910	1.29	2.11E-09	
Putative toxin-antitoxin system protein	LM6179_RS15455	1.24	2.20E-05	
Putative toxin-antitoxin system protein	LM6179_RS15450	1.04	1.84E-05	
conserved membrane protein of unknown function	LM6179_RS15475	0.80	6.92E-04	Yes, upregulated (LMR479A_RS14845)
putative resolvase	LM6179_RS15415	0.79	6.49E-04	
conserved protein of unknown function	LM6179_RS15385	0.74	4.18E-03	
uvrX – putative DNA repair protein	LM6179_RS15380	0.72	2.91E-02	Yes, downregulated (LMR479A_RS15080)
conserved protein of unknown function	LM6179_RS15480	-0.62	5.75E-04	Yes, downregulated (LMR479A_RS14850)
Transposase	LM6179_RS15565	-0.76	2.76E-03	
conserved protein of unknown function	LM6179_RS15445	-0.96	1.62E-05	
conserved protein of unknown function	LM6179_RS15485	-1.09	1.21E-06	
cadC – cadmium efflux system accessory protein Tn*5422*	LM6179_RS15350	-1.10	6.50E-05	Yes, downregulated (LMR479A_RS15130)
conserved protein of unknown function	LM6179_RS15425	-1.27	1.76E-02	
cadA - cadmium transporting ATPase Tn*5422*	LM6179_RS15345	-1.37	4.09E-07	Yes, downregulated (LMR479A_RS15125)
conserved protein of unknown function	LM6179_RS15340	-1.47	1.77E-05	
conserved protein of unknown function	LM6179_RS15460	-1.93	3.02E-16	
Putative HTH transcriptional regulator	LM6179_RS15465	-2.02	1.50E-10	
conserved protein of unknown function	LM6179_RS15330	-2.13	3.63E-08	
conserved protein of unknown function	LM6179_RS15430	-2.18	2.20E-05	
conserved protein of unknown function	LM6179_RS15290	-2.22	3.06E-07	
conserved membrane protein of unknown function	LM6179_RS15320	-2.58	6.55E-12	

Putative typeII/typeIV secretion system protein	LM6179_RS15325	-2.68	6.20E-20	
conserved protein of unknown function	LM6179_RS15335	-2.71	8.04E-07	
Plasmid replication protein *repA*	LM6179_RS15365	-2.72	3.06E-33	Yes, downregulated (LMR479A_RS15065)
conserved protein of unknown function	LM6179_RS15315	-2.75	9.84E-18	
conserved protein of unknown function	LM6179_RS15295	-2.87	3.06E-07	
conserved protein of unknown function	LM6179_RS15310	-3.09	4.09E-14	
conserved protein of unknown function	LM6179_RS15300	-3.61	2.91E-04	
conserved protein of unknown function	LM6179_RS15305	-3.85	1.07E-04	
Predicted protein	LM6179_RS15435	-4.56	2.94E-10	
Predicted protein	LM6179_RS15440	-4.70	2.63E-07	

Supplementary Table S13: Differentially expressed genes on pLMR479a after 30 min exposure to 1% lactic acid. Genes that are also present in pLM6179 are highlighted in grey.

**DE genes, putative function**	**Locus_tag**	**Log2fold change**	**Q value**	**DE gene in pLM6179 1% lactic acid (pLM6179 locus_tag)**
conserved exported protein of unknown function	LMR479A_RS15015	3.84	5.16E-19	
conserved protein of unknown function	LMR479A_RS14955	3.60	3.70E-15	
conserved exported protein of unknown function	LMR479A_RS15030	3.55	2.30E-10	
conserved protein of unknown function	LMR479A_RS14960	3.33	7.14E-13	
Putative riboswitch		3.27	5.16E-19	
conserved exported protein of unknown function	LMR479A_RS15025	3.15	2.42E-04	
conserved protein of unknown function	LMR479A_RS15225	2.70	3.43E-07	
conserved protein of unknown function	LMR479A_RS14855	2.33	3.58E-03	
conserved exported protein of unknown function	LMR479A_RS15045	2.06	4.25E-06	
conserved protein of unknown function	LMR479A_RS14840	1.91	3.09E-07	
Putative multicopper oxidase	LMR479A_RS15145	1.68	2.39E-04	
exported protein of unknown function	LMR479A_RS15090	1.42	3.398E-04	
conserved membrane protein of unknown function	LMR479A_RS14845	1.31	1.17E-03	Yes, upregulated (LM6179_RS15475)
NADH peroxidase	LMR479A_RS15260	-0.93	4.70E-02	
uvrX – putative DNA repair protein	LMR479A_RS15080	-1.35	6.76E-03	Yes, upregulated (LM6179_RS15380)
conserved protein of unknown function	LMR479A_RS15155	-1.48	2.99E-04	
conserved protein of unknown function	LMR479A_RS14850	-1.66	2.14E-04	
SNF2 family protein (fragment)	LMR479A_RS15120	-1.71	4.04E-07	
cadA cadmium-transporting ATPase Tn*5422*	LMR479A_RS15125	-2.07	5.45E-10	Yes, downregulated (LM6179_RS15345)
cadC Cadmium efflux system accessory protein Tn*5422*	LMR479A_RS15130	-2.16	2.05E-08	Yes, downregulated (LM6179_RS15350)
conserved protein of unknown function	LMR479A_RS15060	-2.91	1.35E-05	
DNA topoisomerase (fragment)	LMR479A_RS15220	-3.23	3.74E-04	
protein of unknown function	LMR479A_RS15055	-3.34	1.02E-04	
conserved protein of unknown function	LMR479A_RS15050	-3.69	4.53E-06	
protein of unknown function	LMR479A_RS15100	-3.81	3.67E-11	
Plasmid replication protein *repA*	LMR479A_RS15065	-4.01	6.57E-28	Yes, downregulated (LM6179_RS15365)
Glycine betaine ABC transporter glycine betaine-binding protein GbuC	LMR479A_RS15255	-6.14	9.56E-13	

Supplementary Table 14: BlastN searches of the putative plasmid riboswitch from plasmid pLMR479a against NCBI nr

subject accession no.	Description	% identity	alignment length	query start	query end	subject start	subject end	e-value	bit score
CP041214.1	Listeria monocytogenes strain LMP18-H8393 plasmid	100	109	1	109	19951	19843	2.28E-48	202
KY613776.1	Listeria monocytogenes strain LM-F-33 plasmid	100	109	1	109	5754	5862	2.28E-48	202
KY613770.1	Listeria monocytogenes strain LM-F-123 plasmid	100	109	1	109	72337	72229	2.28E-48	202
KY613765.1	Listeria monocytogenes strain LM-F-75	100	109	1	109	75154	75262	2.28E-48	202
KY613763.1	Listeria monocytogenes strain LM-F-70 plasmid	100	109	1	109	72208	72100	2.28E-48	202
KY613761.1	Listeria monocytogenes strain LM-F-129 plasmid	100	109	1	109	72333	72225	2.28E-48	202
KY613758.1	Listeria monocytogenes strain LM-F-56 plasmid	100	109	1	109	72334	72226	2.28E-48	202
KY613754.1	Listeria monocytogenes strain LM-F-61 plasmid	100	109	1	109	72338	72230	2.28E-48	202
KY613752.1	Listeria monocytogenes strain LM-F-122 plasmid	100	109	1	109	72588	72480	2.28E-48	202
KY613746.1	Listeria monocytogenes strain LM-F-57 plasmid	100	109	1	109	72334	72226	2.28E-48	202
KY613744.1	Listeria monocytogenes strain LM-F-146 plasmid	100	109	1	109	72334	72226	2.28E-48	202
KY613743.1	Listeria monocytogenes strain LM-F-127 plasmid	100	109	1	109	21931	22039	2.28E-48	202
KY613742.1	Listeria monocytogenes strain LM-F-12 plasmid	100	109	1	109	96260	96368	2.28E-48	202
KY613741.1	Listeria monocytogenes strain LM-F-28 plasmid	100	109	1	109	44270	44378	2.28E-48	202
KY613738.1	Listeria monocytogenes strain LM-F-119 plasmid	100	109	1	109	72537	72429	2.28E-48	202
KY613737.1	Listeria monocytogenes strain LM-F-74 plasmid	100	109	1	109	75704	75812	2.28E-48	202
CP025569.1	Listeria monocytogenes strain PIR00540 plasmid	100	109	1	109	29941	29833	2.28E-48	202
CP025561.1	Listeria monocytogenes strain PIR00545 plasmid	100	109	1	109	23765	23657	2.28E-48	202
CP025441.1	Listeria monocytogenes strain MF6172 plasmid pMF6172	100	109	1	109	20114	20222	2.28E-48	202
CP025439.1	Listeria monocytogenes strain MF4697 plasmid pMF4697	100	109	1	109	20114	20222	2.28E-48	202
CP025260.1	Listeria monocytogenes strain MF4624 plasmid pMF4624	100	109	1	109	20114	20222	2.28E-48	202
CP025083.1	Listeria monocytogenes strain MF4626 plasmid pMF4626	100	109	1	109	20116	20224	2.28E-48	202

CP023753.1	Listeria monocytogenes strain AT3E plasmid pLM58	100	109	1	109	20116	20224	2.28E-48	202
CP023051.1	Listeria monocytogenes strain FDA00011238 plasmid	100	109	1	109	28087	28195	2.28E-48	202
CP023053.1	Listeria monocytogenes strain FDA00006905 plasmid	100	109	1	109	7810	7918	2.28E-48	202
CP022021.1	Listeria monocytogenes strain FDA00006907 plasmid pCFSAN021445	100	109	1	109	89374	89482	2.28E-48	202
CP022021.1	Listeria monocytogenes strain FDA00006907 plasmid pCFSAN021445	100	109	1	109	151292	151400	2.28E-48	202
CP019168.1	Listeria monocytogenes strain HPB5622 plasmid	100	109	1	109	48612	48504	2.28E-48	202
CP019166.1	Listeria monocytogenes strain HPB5415 plasmid	100	109	1	109	48612	48504	2.28E-48	202
KX467250.1	Listeria monocytogenes strain LM-C-273 plasmid	100	109	1	109	5754	5862	2.28E-48	202
CP015985.1	Listeria monocytogenes strain 2015TE24968 plasmid	100	109	1	109	2299	2191	2.28E-48	202
KU513859.1	Listeria monocytogenes strain IZSAM_Lm_15_17439_A14	100	109	1	109	81765	81657	2.28E-48	202
CP014251.1	Listeria monocytogenes strain CFSAN010068 plasmid	100	109	1	109	21147	21039	2.28E-48	202
CP013725.1	Listeria monocytogenes strain Lm N1546 plasmid	100	109	1	109	22616	22724	2.28E-48	202
HG813248.1	Listeria monocytogenes R479a plasmid pLMR479a	100	109	1	109	81765	81657	2.28E-48	202
CP006611.1	Listeria monocytogenes strain N1-011A plasmid	100	109	1	109	2305	2197	2.28E-48	202
CP006611.1	Listeria monocytogenes strain N1-011A plasmid	100	109	1	109	59861	59753	2.28E-48	202
CP006595.1	Listeria monocytogenes strain R2-502 plasmid	100	109	1	109	51149	51041	2.28E-48	202
FR667692.1	Listeria monocytogenes serotype 1-2b str. SLCC2755	100	109	1	109	20575	20683	2.28E-48	202
CP001603.1	Listeria monocytogenes 08-5578 plasmid pLM5578	100	109	1	109	69513	69621	2.28E-48	202
AL592102.1	Listeria innocua Clip11262 plasmid pLI100	100	109	1	109	43652	43760	2.28E-48	202
NZ_CP045973.1	Listeria monocytogenes strain AUSMDU00000224 plasmid	100	109	1	109	20246	20354	2.28E-48	202
NZ_CP015985.1	Listeria monocytogenes strain 2015TE24968 plasmid pl2015TE24968	100	109	1	109	2299	2191	2.28E-48	202
KC980924.1	Listeria monocytogenes strain 11GZL18	100	33	77	109	33727	33695	4.04E-06	62.1
